# Supplementary material for: Uneven Missing Data Skew Phylogenomic Relationships within the Lories and Lorikeets
Source: Genome Biol Evol. 2020 May 29;12(7):1131–47. doi: 10.1093/gbe/evaa113 (PMC7486955; doi:10.1093/gbe/evaa113)
Supplement: evaa113_Supplementary_Data [file evaa113_supplementary_data.zip › Supplementary_Table_S4.pdf]

Supplementary Table S4. Neural network model output for subclades that assessed predictors of Δ locus-wise log-likelihood scores. Shown are the variable importance of the five alignment statistics included in the neural net model for each subclade in Loriini. a) *Chalcopsitta* and *Pseudeos*, b) *Charmosyna*, *Vini*, and *Phigys*, c) *Eos*, *Trichoglossus*, *Glossopsitta concinna*, and *Psitteuteles iris*, d) *Parvipsitta* and *Psitteuteles*, and e) *Lorius*, f) *Neopsittacus*. The per locus statistics were as follows:parsimony informative sites (PIS), alignment length (Ali. Len.), number of variables sites (Var. Sites), number of undetermined characters (Undet. Char.), and GC content (GC. Cont.). The sample size for each subclade was the total number of alignments and 75%/25% was used to train/test each model. Reported are R2and mean square error (MSE).

**Low Coverage**	**PIS**	**Ali. Len.**	**Var. Sites**	**Undet. Char.**	**GC Cont.**	**R2 (SD)**	**RMSE (SD)**
Chalcopsitta/Pseudeos	52.23	12.12	15.53	12.50	7.62	0.008 (0.007)	0.063 (0.003)
Charmosyna/Vini/Phigys	41.25	13.34	22.36	6.87	16.18	0.021 (0.014)	0.061 (0.002)
Eos/Trichoglossus/ Glossopsitta/Psitteuteles	52.57	12.54	22.99	8.30	3.59	0.088 (0.100)	0.017 (0.007)
Parvipsitta/Psitteuteles	34.86	9.00	16.71	23.98	15.45	0.021 (0.014)	0.033 (0.005)
Lorius	37.65	17.19	17.53	8.64	19.00	0.026 (0.026)	0.036 (0.003)
Neopsittacus	22.83	32.78	10.64	23.19	10.57	0.002 (0.002)	0.045 (0.003)
**Filtered**	**PIS**	**Ali. Len.**	**Var. Sites**	**Undet. Char.**	**GC Cont.**	**R2 (SD)**	**RMSE (SD)**
Chalcopsitta/Pseudeos	53.86	5.71	16.96	6.34	17.12	0.009 (0.007)	0.030 (0.006)
Charmosyna/Vini/Phigys	20.58	5.96	34.77	11.28	27.41	0.001 (0.001)	0.049 (0.003)
Eos/Trichoglossus/ Glossopsitta/Psitteuteles	60.72	7.00	21.74	4.07	6.47	0.040 (0.035)	0.046 (0.003)
Parvipsitta/Psitteuteles	7.61	22.41	61.15	5.55	3.28	0.090 (0.020)	0.065 (0.002)
Lorius	51.24	6.40	19.73	10.50	12.14	0.006 (0.006)	0.028 (0.005)
Neopsittacus	15.28	15.83	34.42	12.97	21.51	0.011 (0.016)	0.017 (0.009)
