# Supplementary material for: Uneven Missing Data Skew Phylogenomic Relationships within the Lories and Lorikeets
Source: Genome Biol Evol. 2020 May 29;12(7):1131–47. doi: 10.1093/gbe/evaa113 (PMC7486955; doi:10.1093/gbe/evaa113)
Supplement: evaa113_Supplementary_Data [file evaa113_supplementary_data.zip › SUPPLEMENTARY FIG AND TABLE LEGENDS.docx]

**SUPPLEMENTARY TABLE LEGENDS**

**Supplementary Table S1. Metadata for Loriini samples used in this study.**

**Supplementary Table S2. Read and locus statistics.**

**Supplementary Table S3. Mean coverage of each site along a locus.**

**Supplementary Table S4. Neural network model output for subclades that assessed predictors of Δ locus-wise log-likelihood scores** Shown are the variable importance of the five alignment statistics included in the neural network model for each subclade in Loriini. a) *Eos*, *Trichoglossus*, *Glossopsitta concinna*, and *Psitteuteles iris*, b) *Parvipsitta* and *Psitteuteles*, c) *Neopsittacus*, d) *Chalcopsitta* and *Pseudeos*, e) *Lorius*, and f) *Charmosyna*, *Vini*, and *Phigys*. The per locus statistics were as follows: parsimony informative sites (PIS), alignment length (Ali. Len.), number of variables sites (Var. Sites), number of undetermined characters (Undet. Char.), and GC content (GC. content). The sample size for each subclade was the total number of alignments and 75%/25% was used to train/test each model. Reported are R^2^ and mean square error (MSE).

**SUPPLEMENTARY FIGURE LEGENDS**

**Supplementary Figure S1. Sampling map of lory and lorikeet taxa used in this study.** Colored symbols represent material that came from historical (blue) and modern (red) samples.

**Supplementary Figure S2. Plot showing that older samples have more missing data.** Shown is the year the sample was collected at the percentage of missing characters as determined by IQ-TREE in the Filtered datatset. Mean regression line and 95% CI are shown (Adjusted R^2^ = 0.31; *n* = 144; p-value < 0.0001). Not all samples were included in this analysis because accurate ages of the specimens were unavailable.

**Supplementary Figure S3. Low Coverage maximum likelihood trees.** The tree was inferred from a concatenated alignment with all loci (fig. S3A) and an increasing number of loci excluded in increments of the Δ locus-wise log-likelihood scores of: (Δ l-lk) thresholds of: >20 (fig. S3B), >10 (fig. S3C), >2 (fig. S3D), <-10 (fig. S3E), and <-2 (fig. S3F). On each node are shown bootstrap values. Tip labels are colored according to whether the sample came from a historical (blue) or modern (red) sample.

**Supplementary Figure S4. Filtered maximum likelihood trees.** The tree was inferred from a concatenated alignment with all loci (fig. S4A) and an increasing number of loci excluded in increments of the Δ locus-wise log-likelihood scores of: (Δ l–lk) thresholds of: >20 (fig. S4B), >10 (fig. S4C), >2 (fig. S4D), <-10 (fig. S4E), and <-2 (fig. S4F). Bootstrap values are shown on each node. Tip labels are colored according to whether the sample came from a historical (blue) or modern (red) sample.

**Supplementary Figure S5. Low Coverage and Filtered maximum likelihood trees with all samples (*N* = 171) and tips colored by sequencing plate.** Presented are Low Coverage (fig. S5A) and Filtered (fig. S5B) trees where tips were colored according to one of three (Plate 1: red; Plate 2: orange; and Plate 3: blue) lanes, which were sequenced independently over the course of three years.

**Supplementary Figure S6. Low Coverage and Filtered maximum likelihood trees with all samples (*N* = 171) and tips colored according to sample type.** Presented are Low Coverage (fig. S6A) and Filtered (fig. S6B) trees where tips are colored according to whether the sample type came from a historical specimen (blue) or modern tissue (red).

**Supplementary Figure S7. Plot showing the best-fit nucleotide substitution model and the Δ l–lk score for that locus.** Δ l–lk scores were estimated from outlier analysis comparing the topologies of the Low Coverage and Filtered topologies and the Low Coverage alignment. Best-fit nucleotide substitution models were estimated in IQTREE.

**Supplementary Figure S8. Likelihood plots showing locus Δ locus-wise log-likelihoods for topologies estimated with and without missing data for the Filtered dataset.** The y-axis is the Δ locus-wise log-likelihood and the x-axis represents individual loci across the full alignment. Shown are the results for six subclades assessed within Loriini using the Filtered dataset: A) ​*Parvipsitta* and ​*Psitteuteles​*, B) *Chalcopsitta* and ​*Pseudeos*​, C) ​Neopsittacus​, D) ​*Charmosyna*​, *Vini*, and *Phigys*, E) ​*Eos*​, *​Trichoglossus​*, *Glossopsitta concinna*, and ​*Psitteuteles iris*​, and F) ​*Lorius*. Points are colored according to the magnitude of the Δ locus-wise log-likelihood scores using a gradient ranging from > 20 (blue) through < -10 (orange).

**Supplementary Figure S9. Low Coverage maximum likelihood trees where 99.9% of parsimony informative sites in five modern individuals were converted to missing data.** Shown are 11 trees where the sequence was not manipulated in tree A and trees B–K were. Tips are colored according to whether the sample type came from a historical specimen (blue) or modern tissue (red), and black samples are the ones that were manipulated (*Trichoglossus rubritorquis* KU22839, *T. chlorolepidotus* DOT2422, *T. ornatus* DOT7930, *Phigys solitarius* KU22543, and *Charmosyna placentis pallidior* DOT20055). On each node are shown rapid bootstrap values.

**Supplementary Figure S10. Low Coverage maximum likelihood trees sites excluded based on their** Δ s–lk score. Shown trees that were estimated from the exclusion of outlier sites in the Low Coverage alignment that were detected in the comparison of the Low Coverage and Filtered topologies. Trees were inferred from a concatenated alignment with all loci (fig. S10A) and an increasing number of sites excluded in increments of the Δ sites-wise log-likelihood scores of: (Δ s-lk) thresholds of: >20 (fig. S10B), >10 (fig. S10C), >2 (fig. S10D), <-10 (fig. S10E), and <-2 (fig. S10F). On each node are shown bootstrap values. Tip labels are colored according to whether the sample came from a historical (blue) or modern (red) sample.

**Supplementary Figure S11. Low Coverage maximum likelihood trees with varying levels of data completeness.** Shown are 11 trees (A–K) estimated from alignments where the percentage of data completeness ranged from 0% (tree A; all sites were used) through 100% (tree K; all sites with missing data were excluded). In increments of 10%, each successive tree had higher data completeness. At each site along the alignment a specified percentage of individuals were required to have the site in order to be retained in the alignment. The trees are as follows and the percentages refer to the level of data completeness–A:0%; B:10%; C:20%; D:30%; E:40%; F:50%; G:60%; H:70%; I:80%; J:90%; and K:100%. Tips are colored according to whether the sample type came from a historical specimen (blue) or modern tissue (red).
