# Supplementary material for: Uneven Missing Data Skew Phylogenomic Relationships within the Lories and Lorikeets
Source: Genome Biol Evol. 2020 May 29;12(7):1131–47. doi: 10.1093/gbe/evaa113 (PMC7486955; doi:10.1093/gbe/evaa113)
Supplement: evaa113_Supplementary_Data [file evaa113_supplementary_data.zip › Supplementary_Figures.pdf]

Fig. S1: Sampling Map

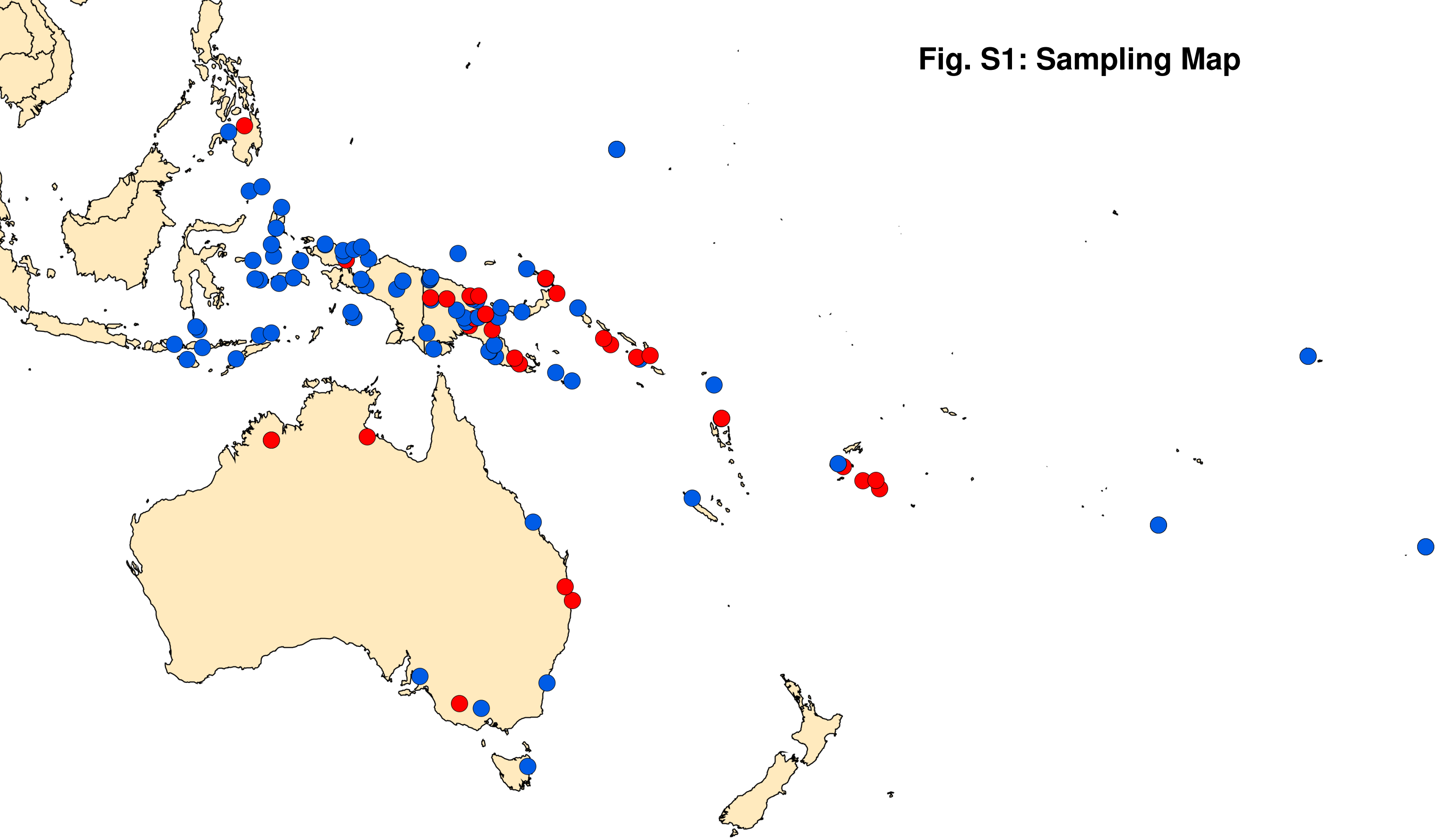

Fig. S2: Percentage of Missing Data versus Age of Specimen

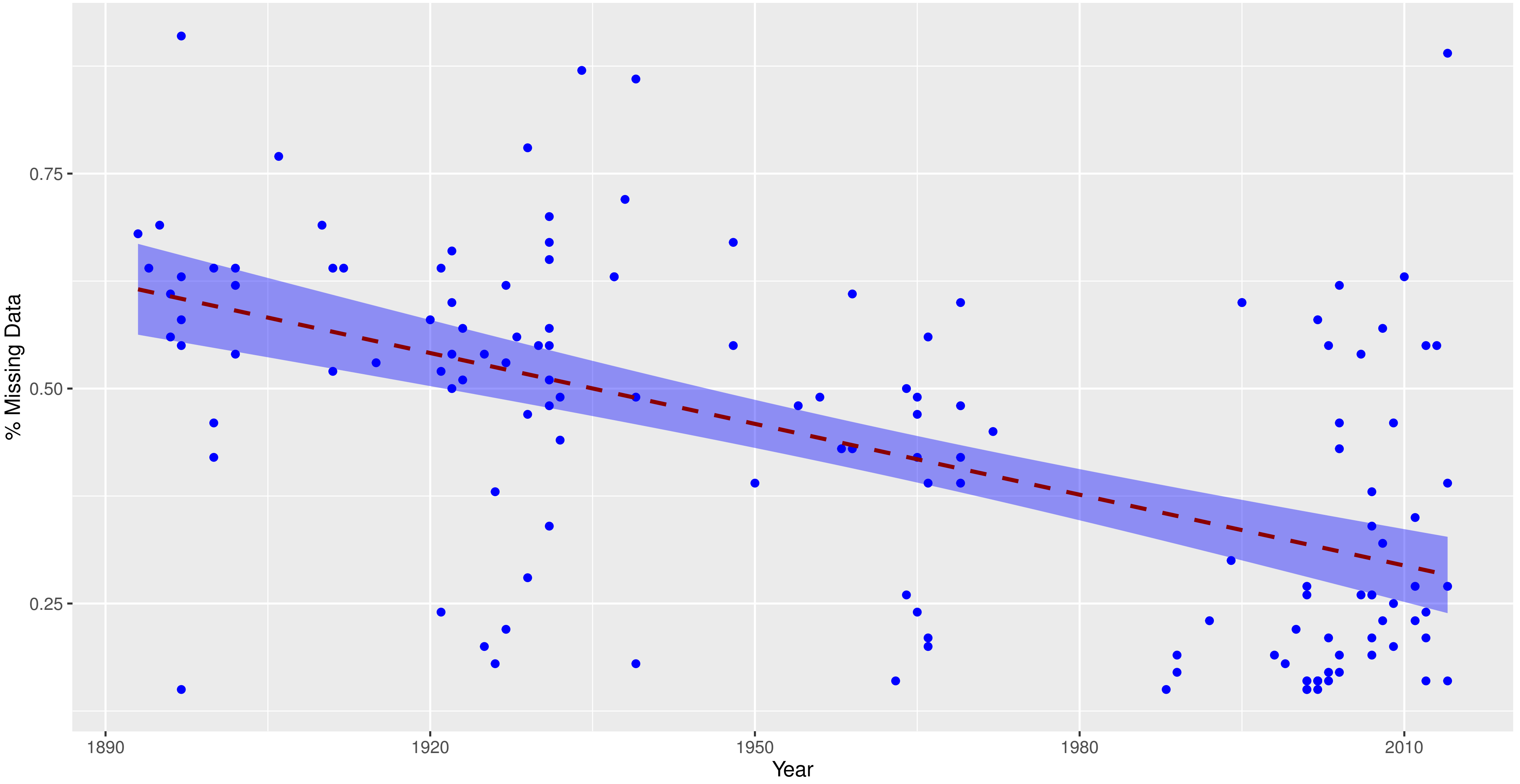

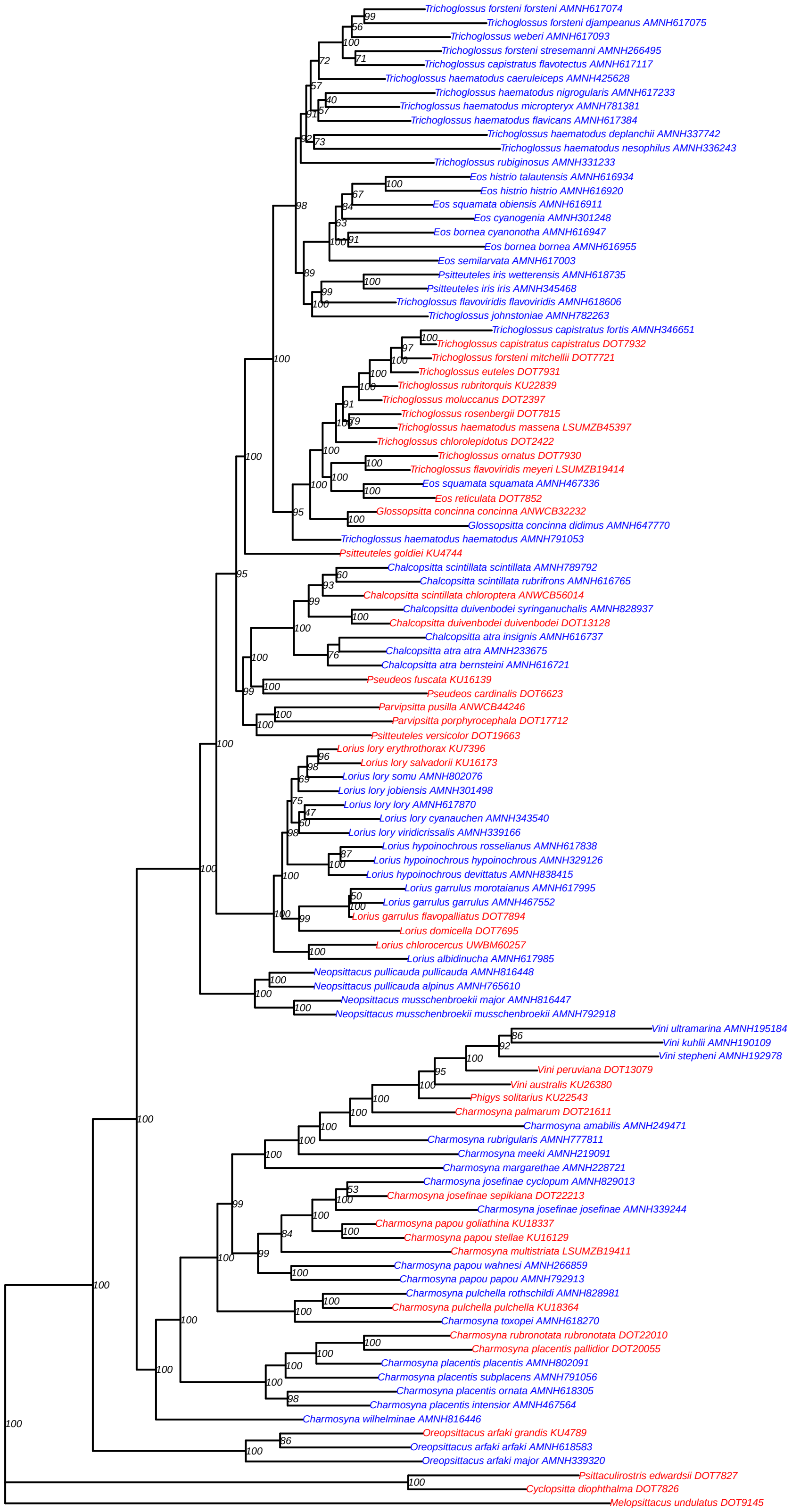

Fig. S3A) Low Coverage: All Loci

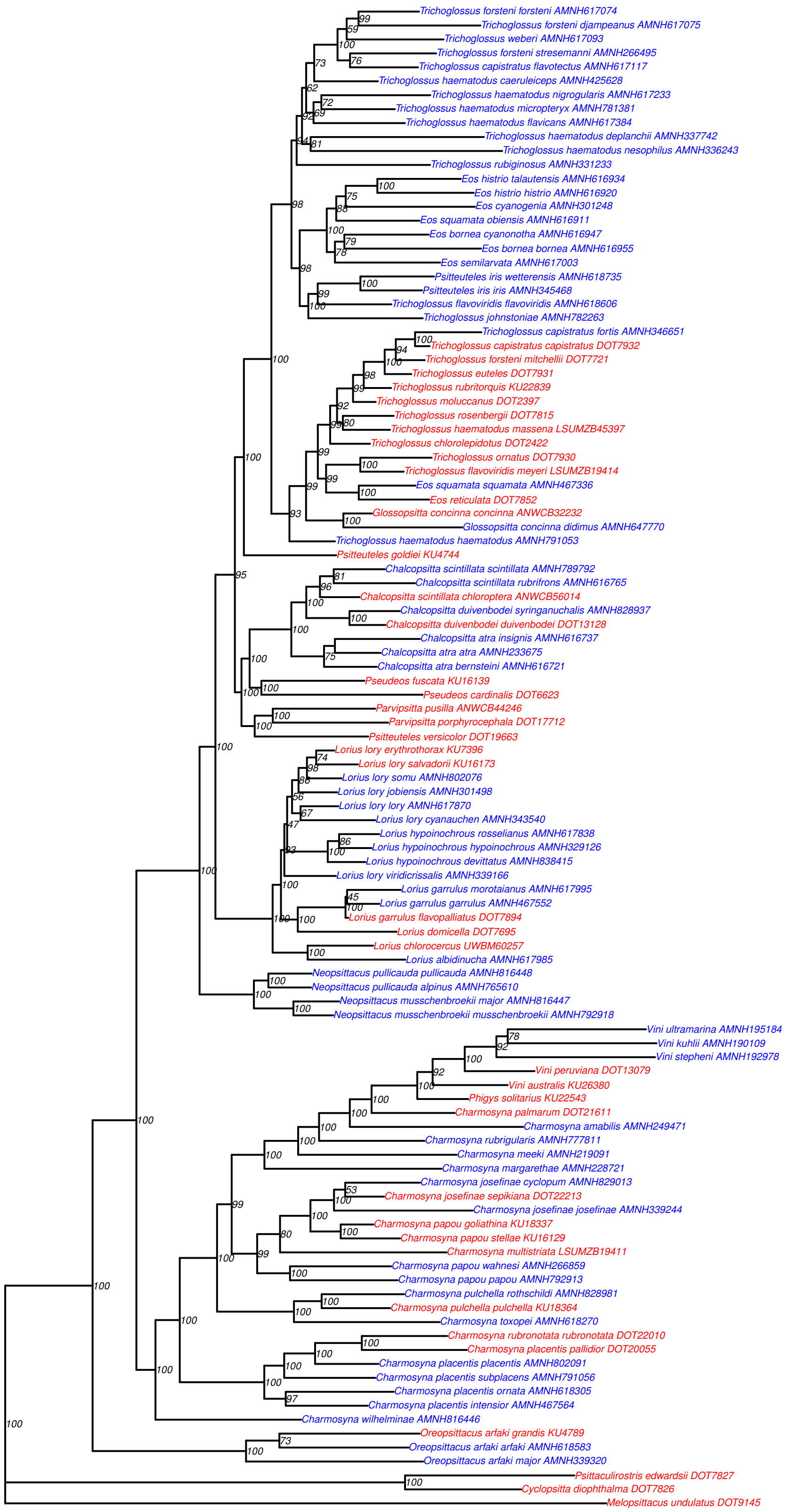

Fig. S3B) Low Coverage: Δ l–lk > 20 Excluded

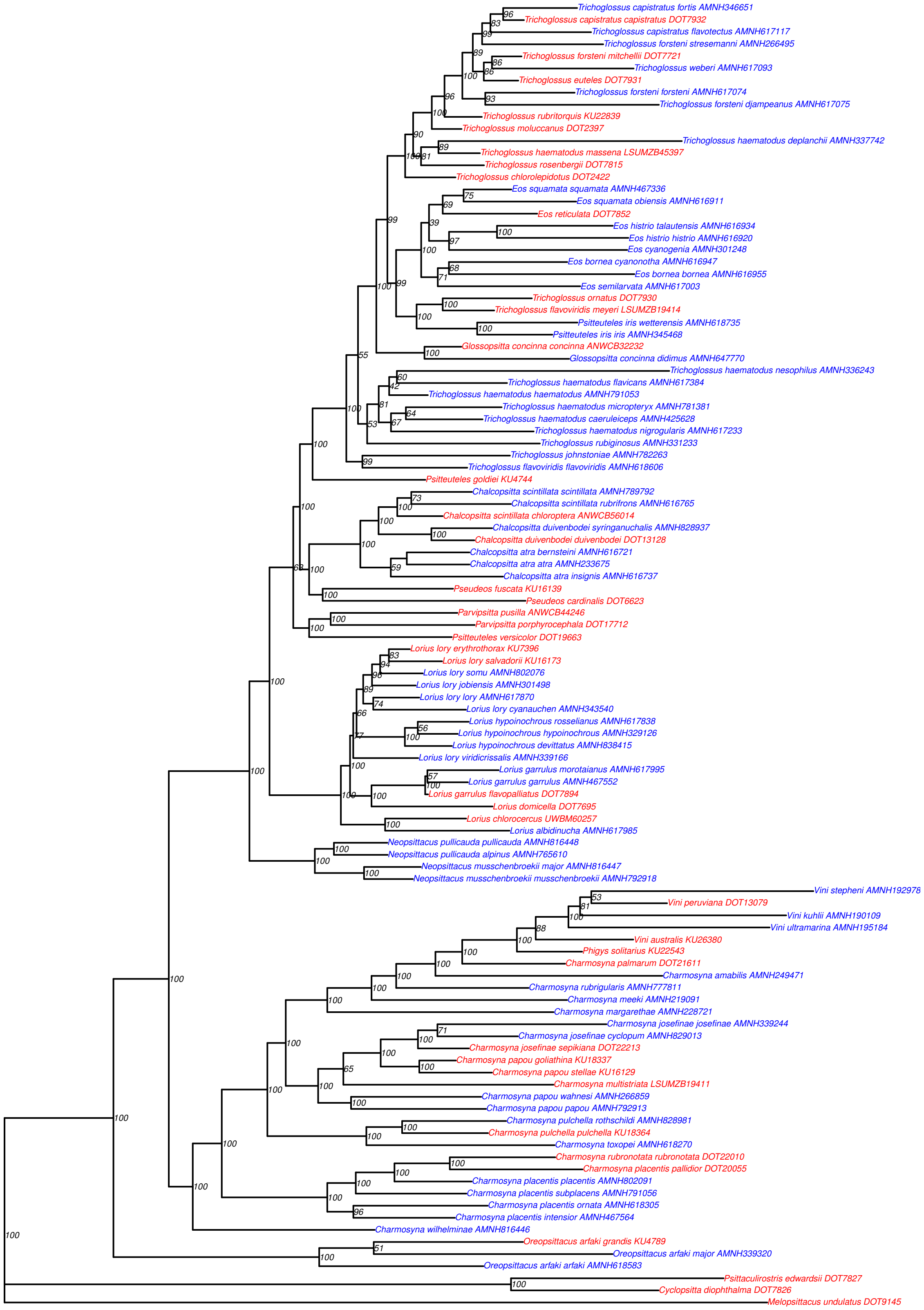

Fig. S3C) Low Coverage: Δ l–lk > 10 Excluded

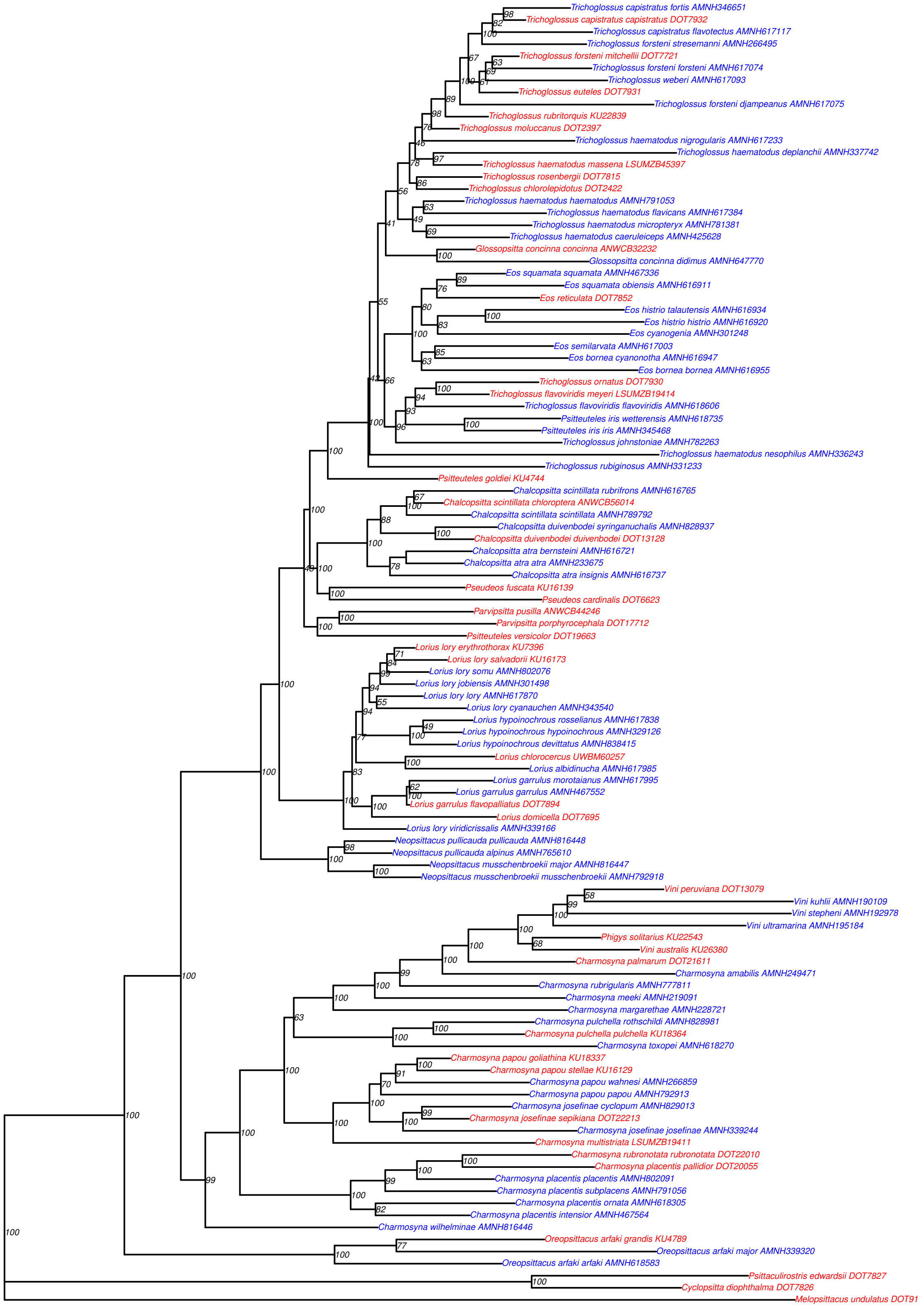

Fig. S3D) Low Coverage: Δ l–lk > 2 Excluded

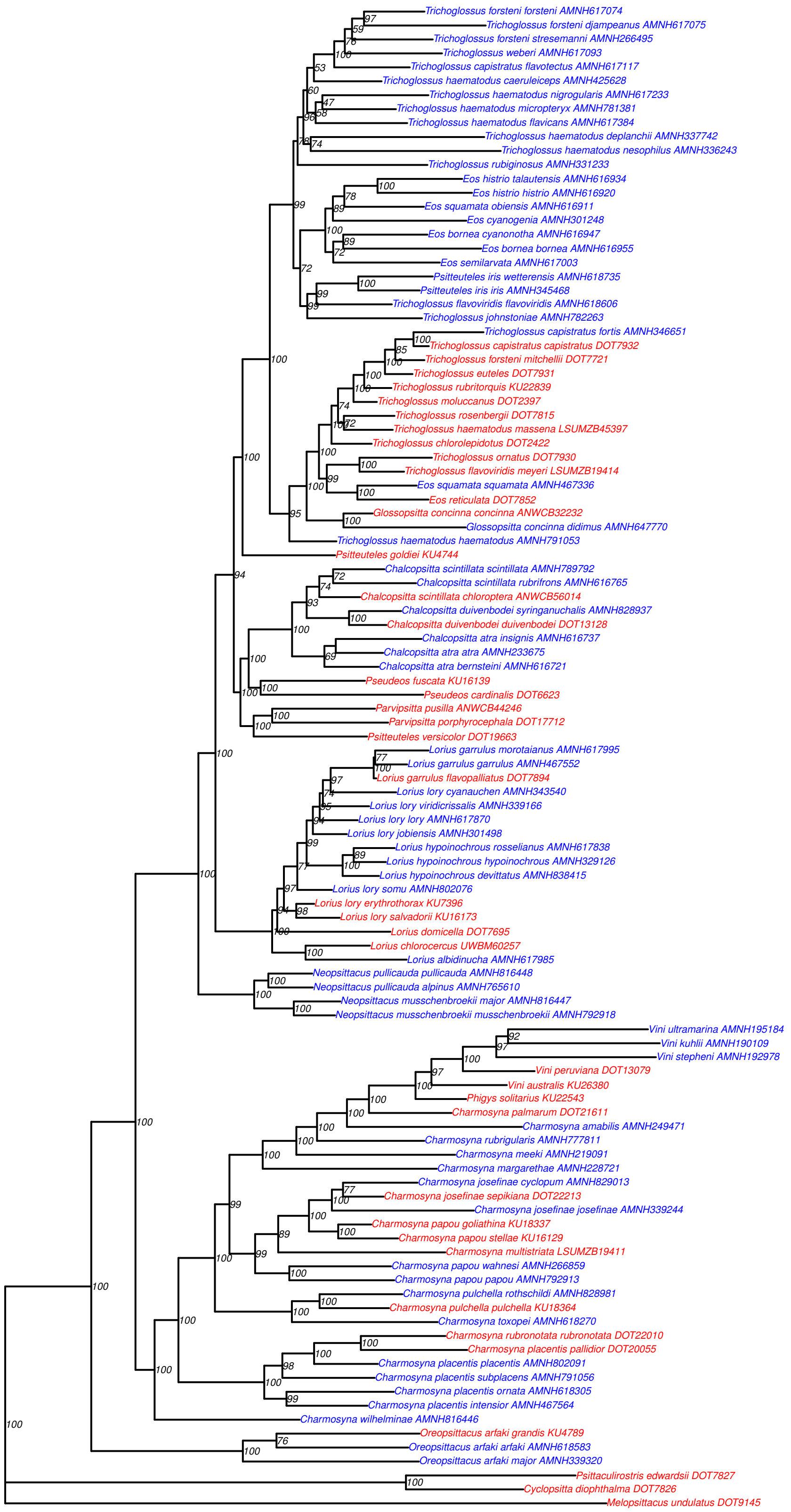

Fig. S3E) Low Coverage: Δ l–lk < −10 Excluded

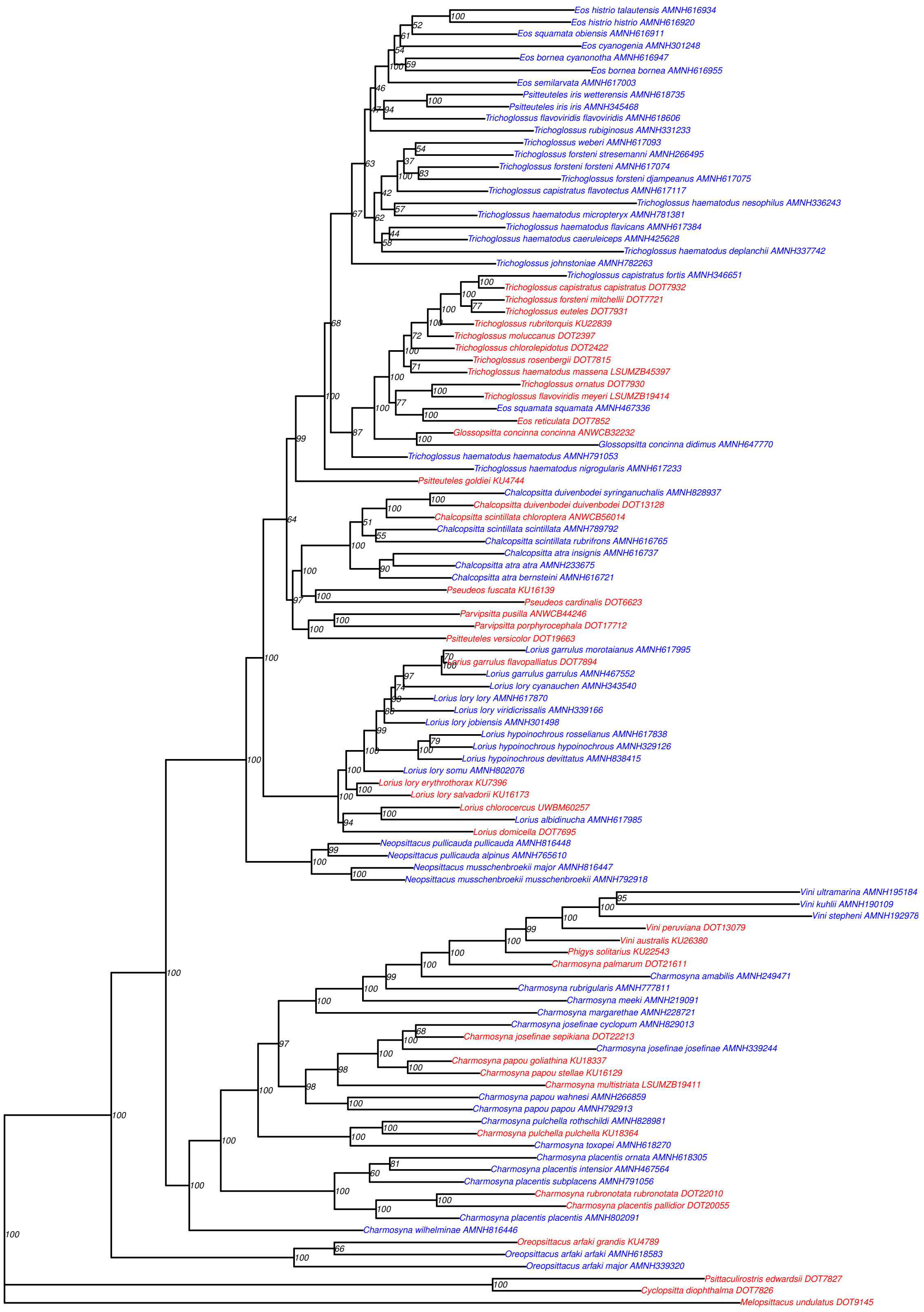

Fig. S3F) Low Coverage: Δ l–lk < –2 Excluded

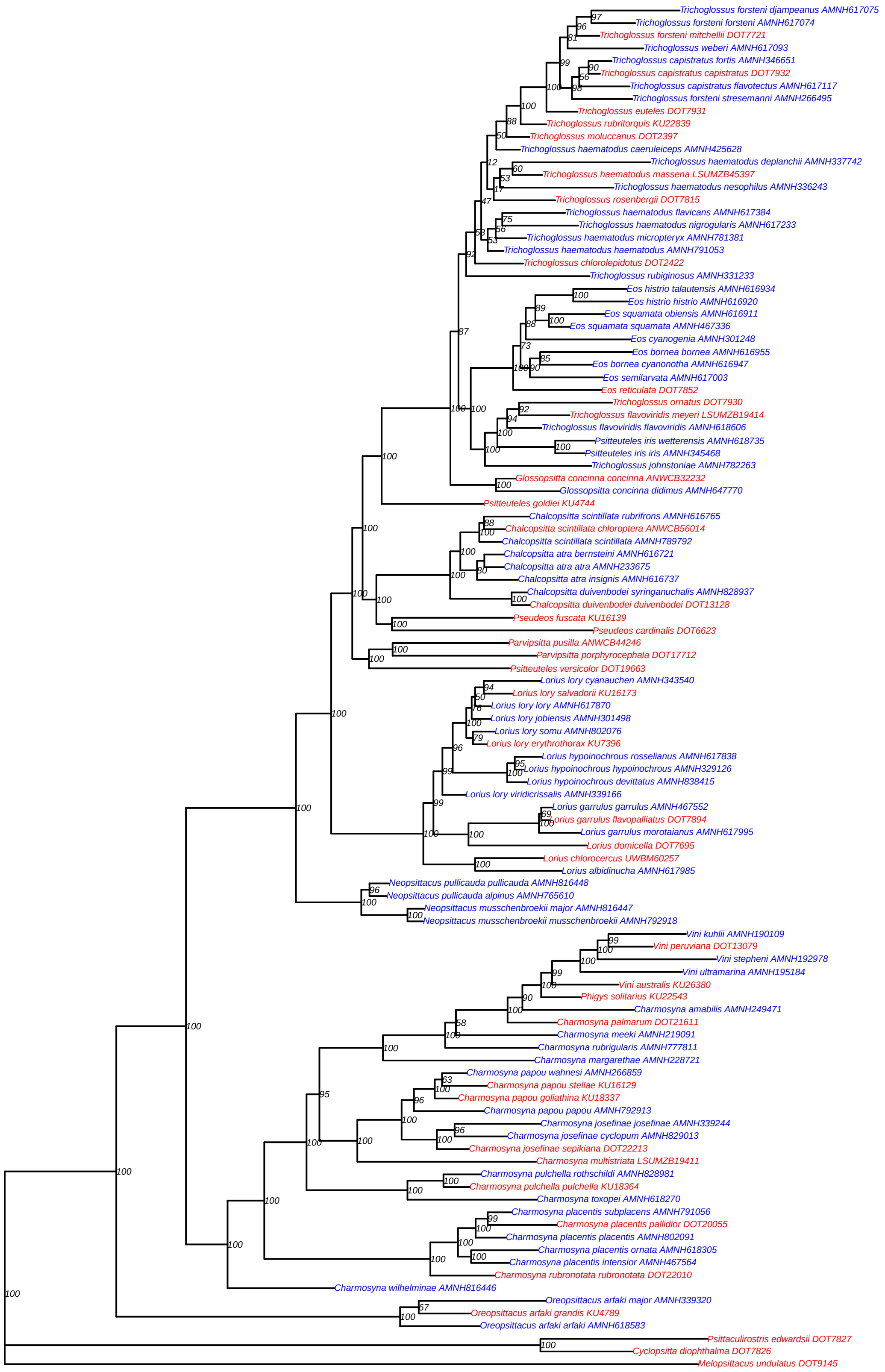

Fig. S4A) Filtered: All Loci

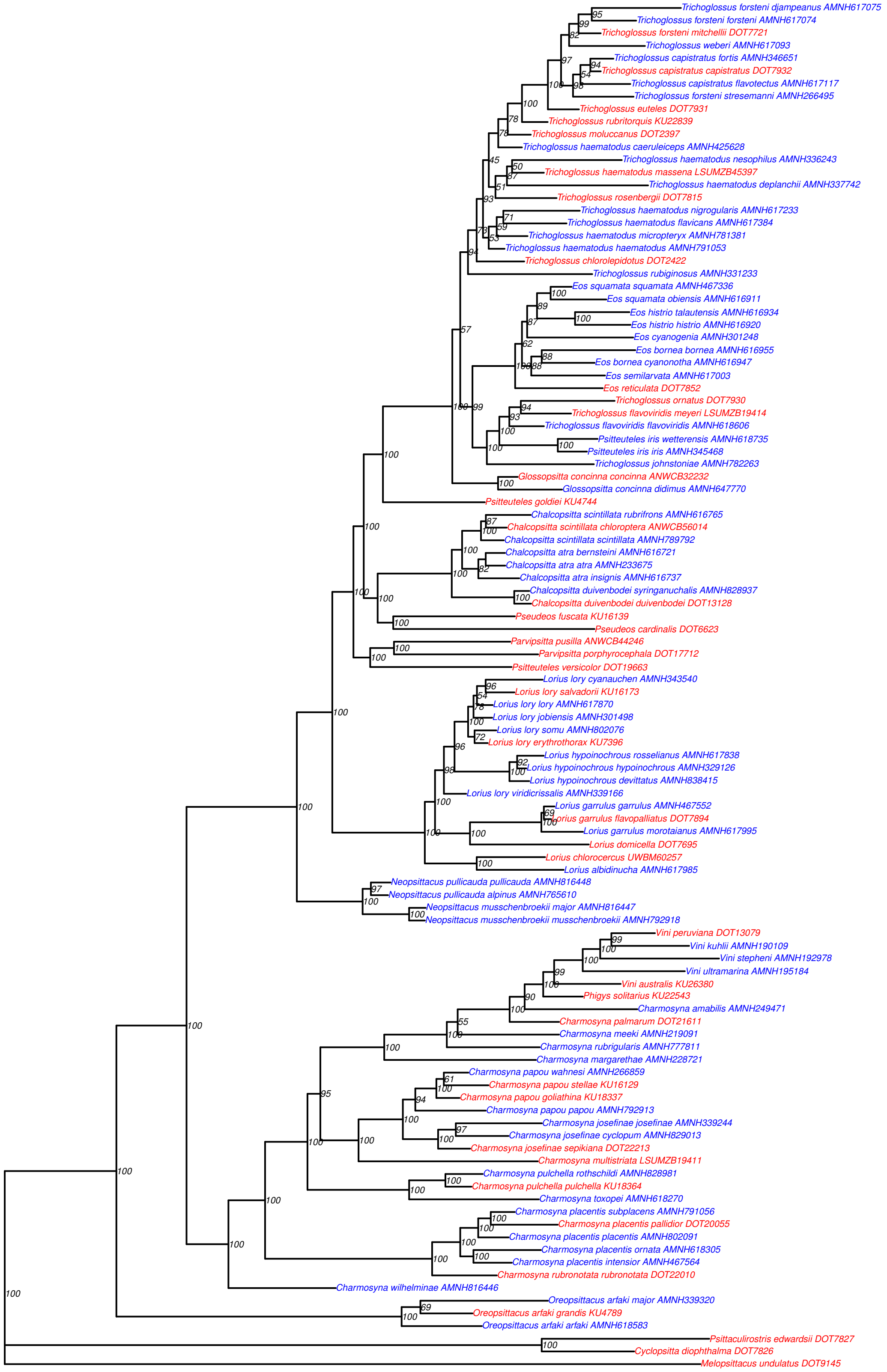

Fig. S4B) Filtered: Δ l–lk > 20 Excluded

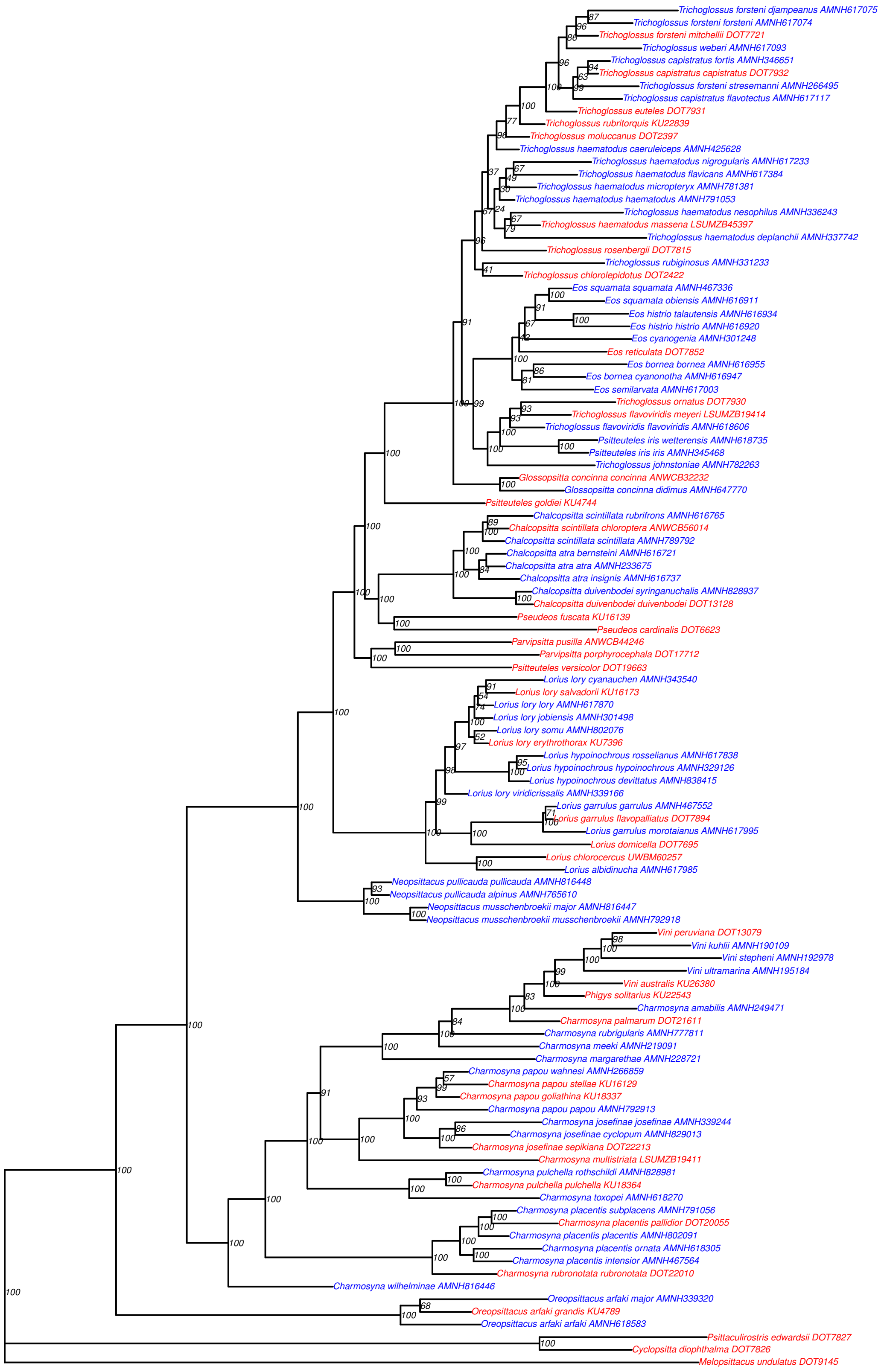

Fig. S4C) Filtered: Δ l–lk > 10 Excluded

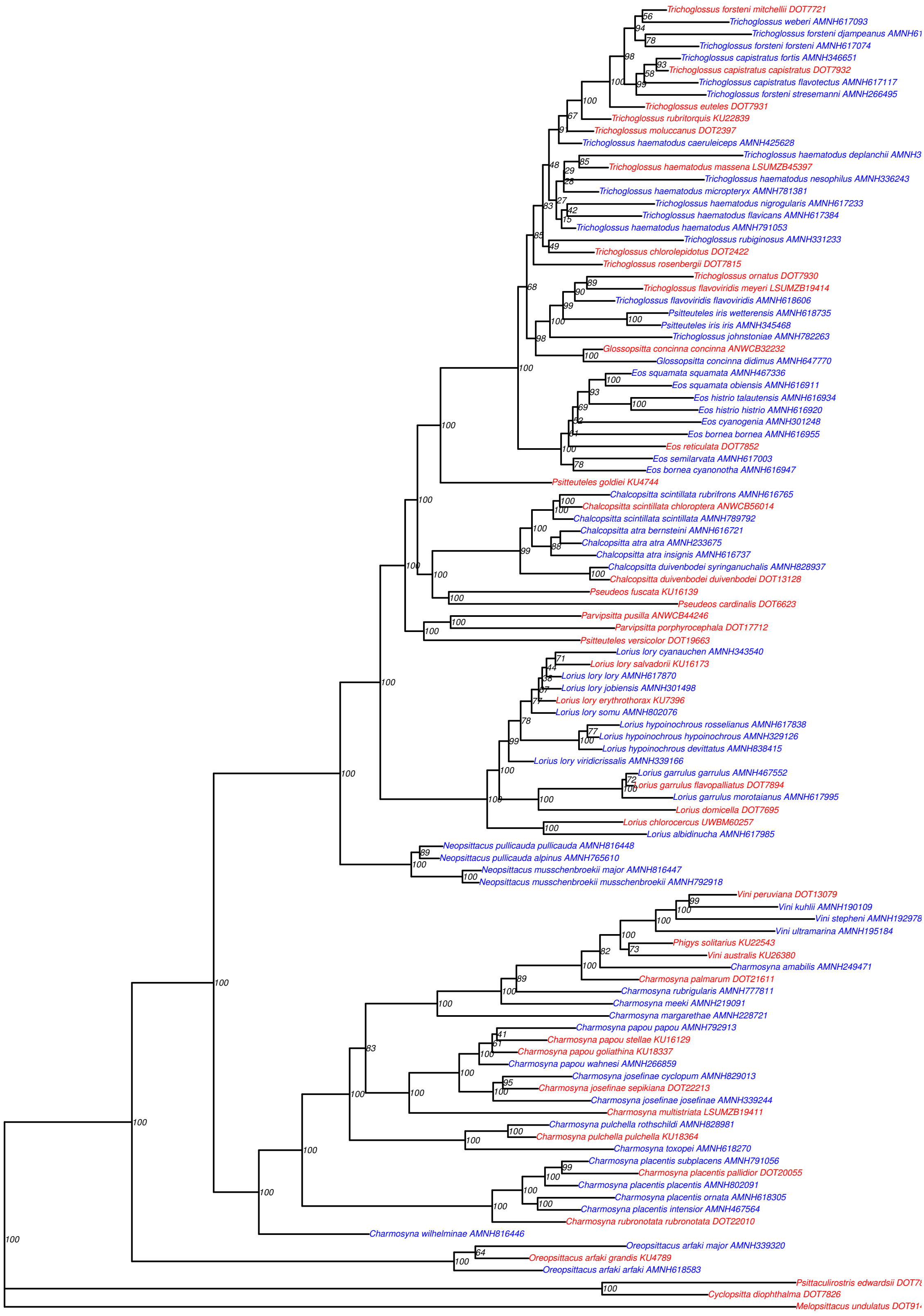

Fig. S4D) Filtered: Δ l–lk > 2 Excluded

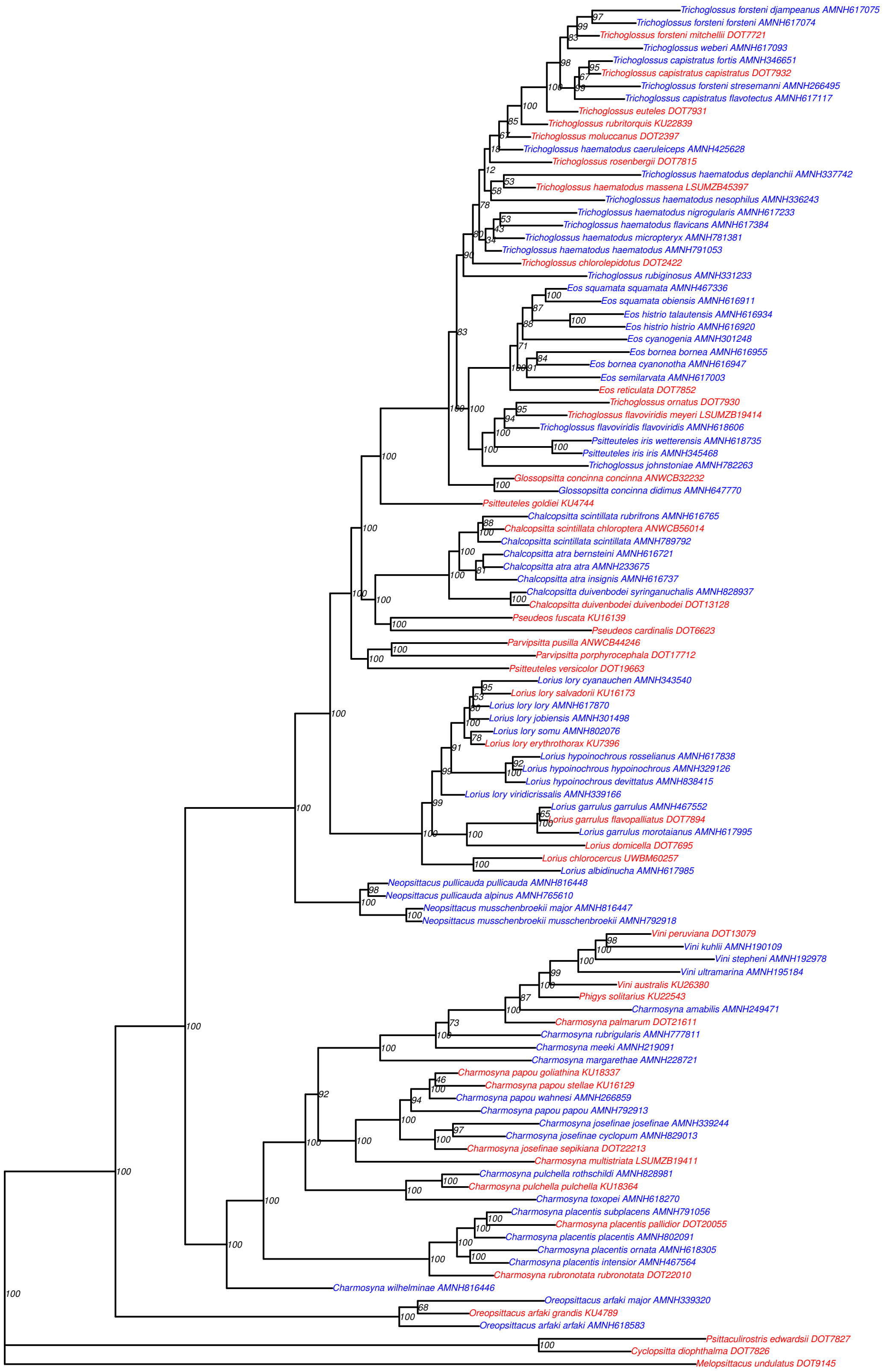

Fig. S4E) Filtered: Δ l–lk < –10 Excluded

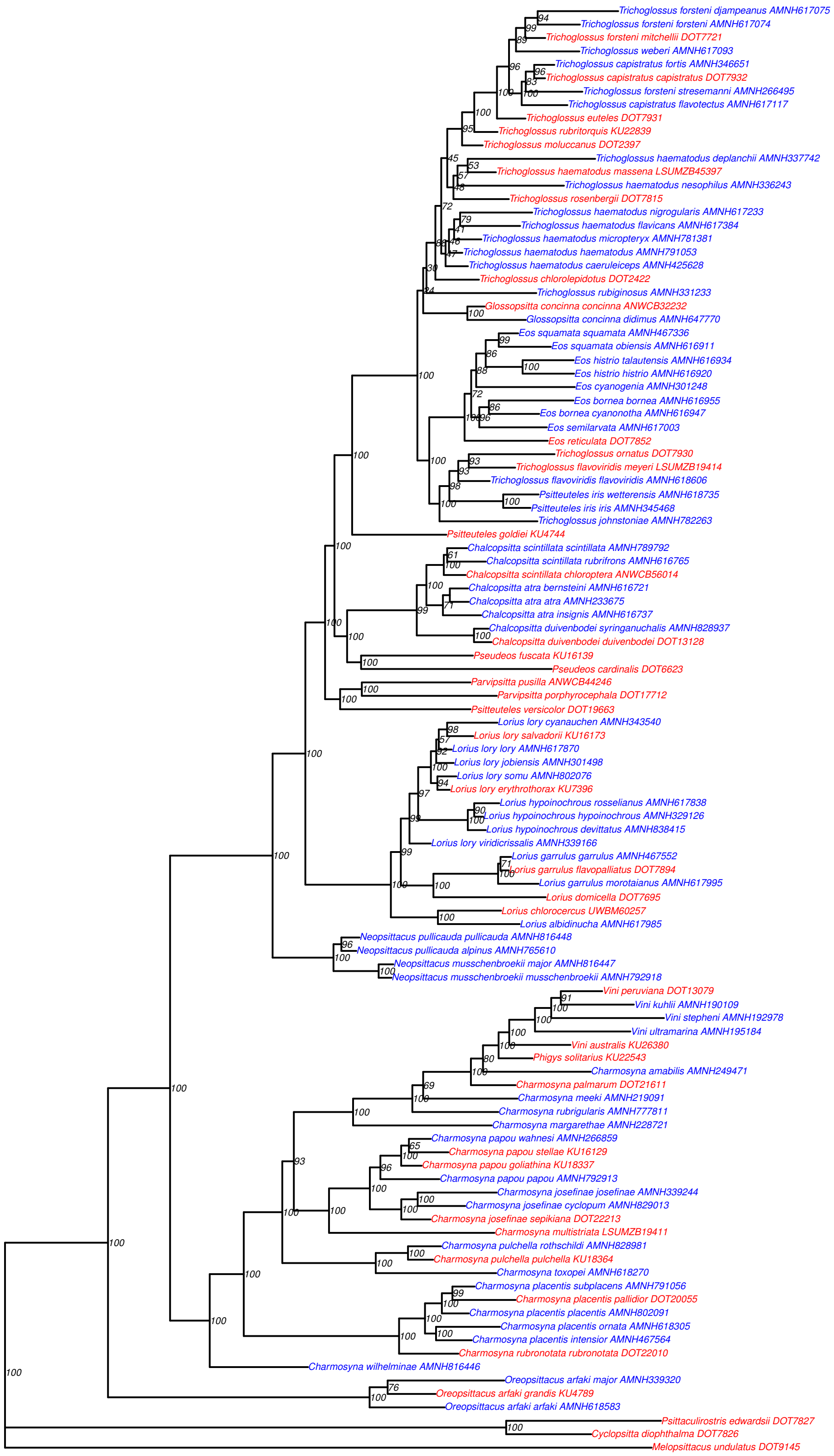

Fig. S4F) Filtered: Δ l–lk < –2 Excluded

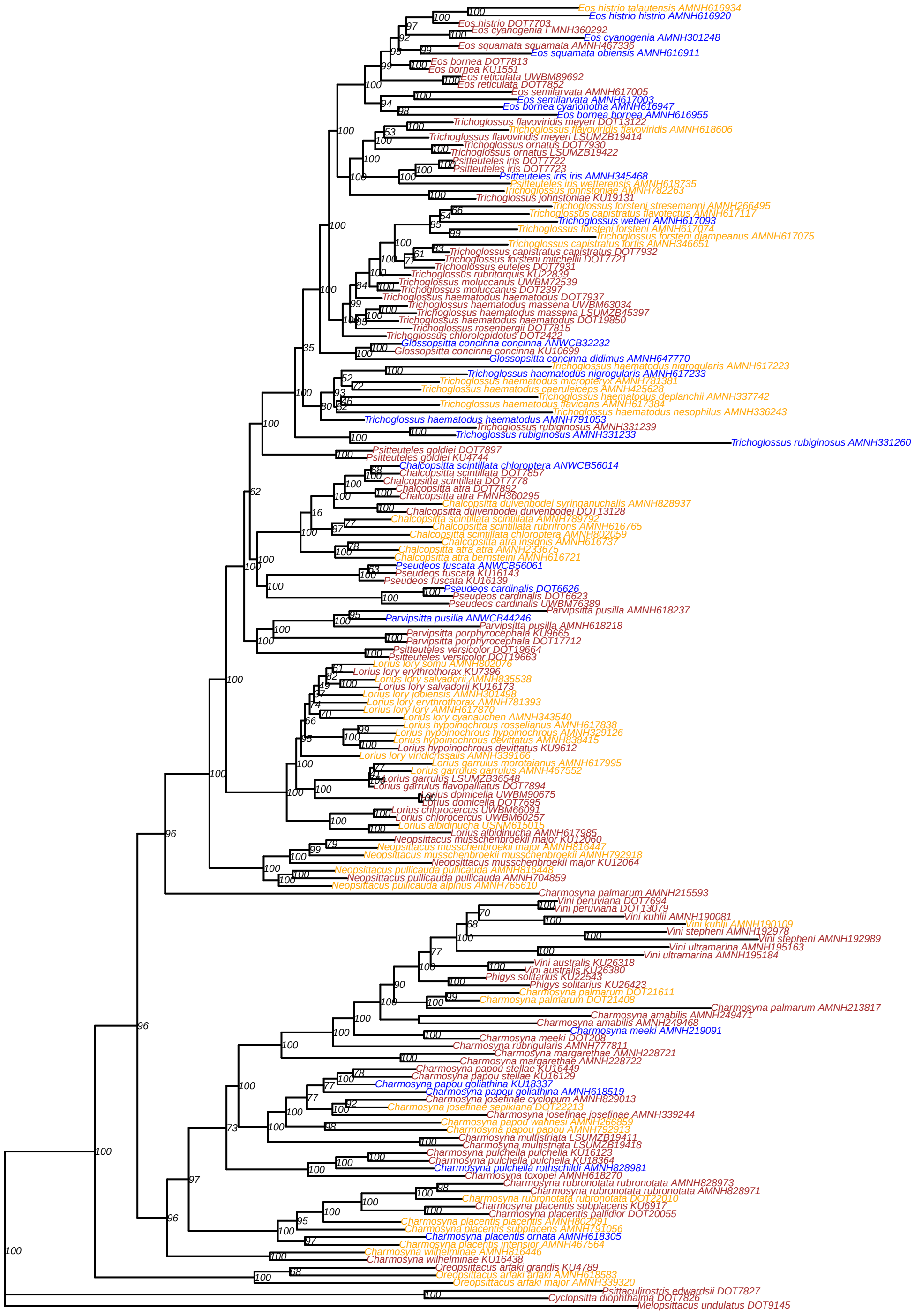

Fig. S5A) Low Coverage: Batch Effect

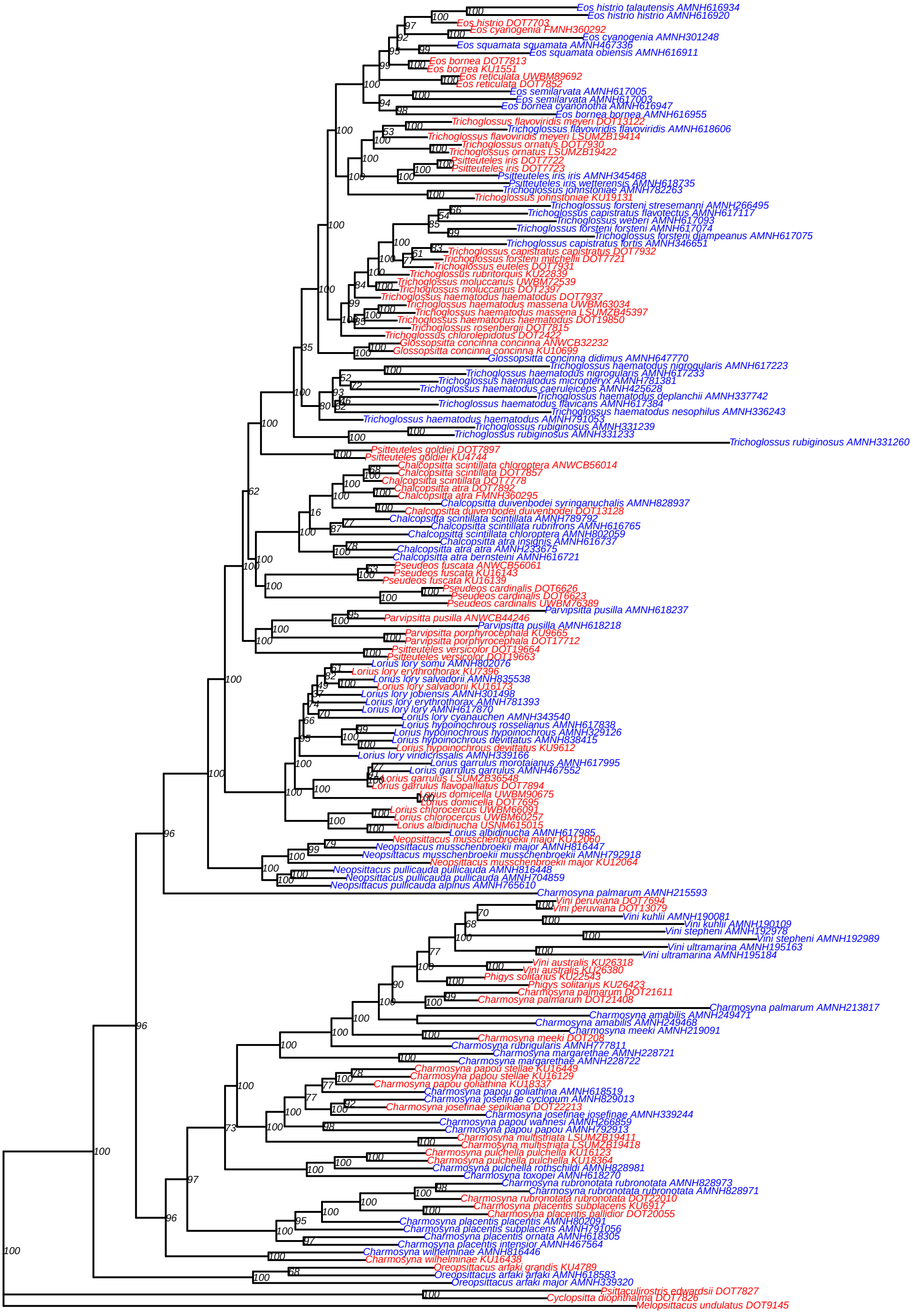

Fig. S5B) Low Coverage: Sample Type

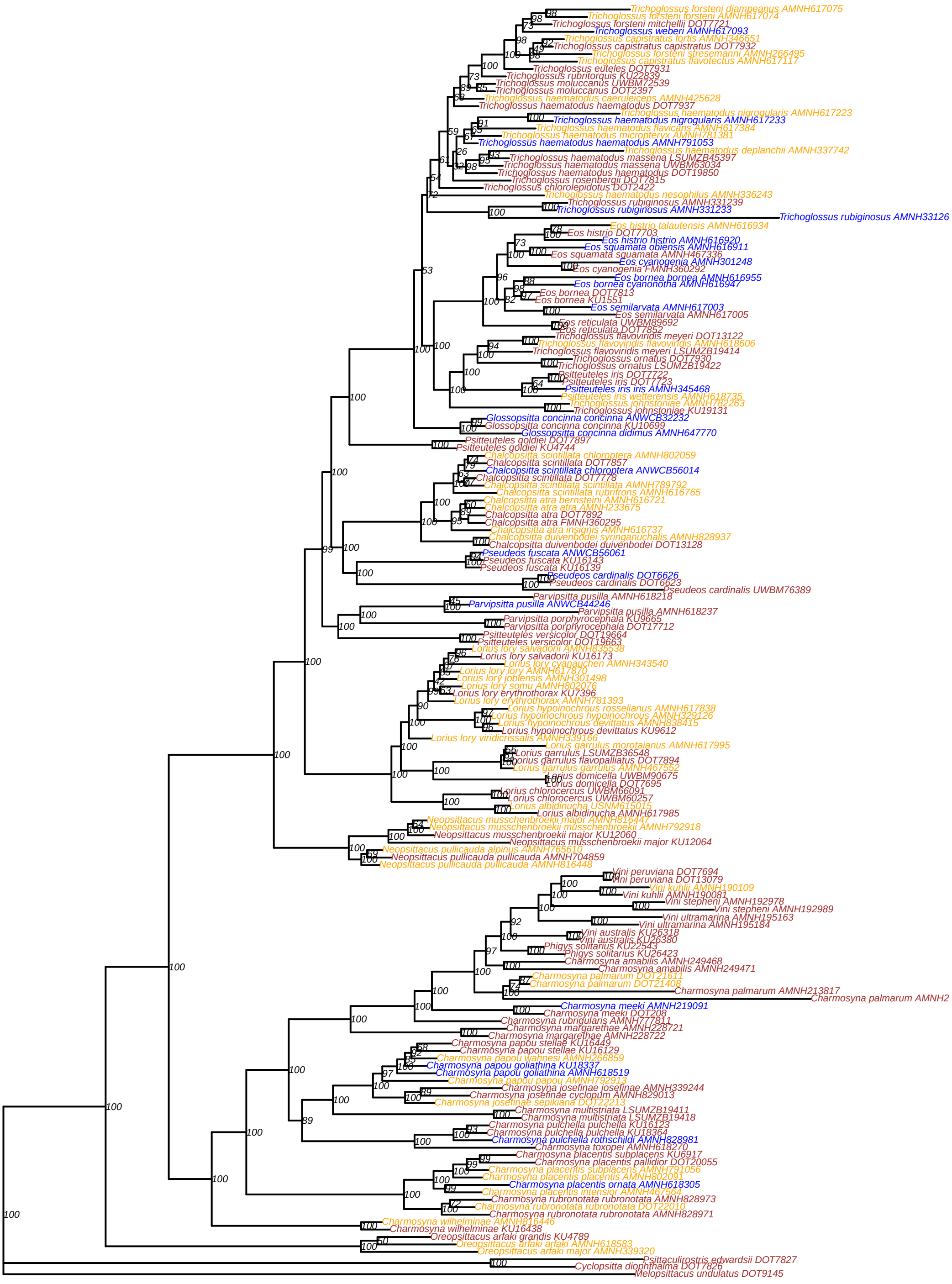

Fig. S6A) Low Coverage: Batch Effect

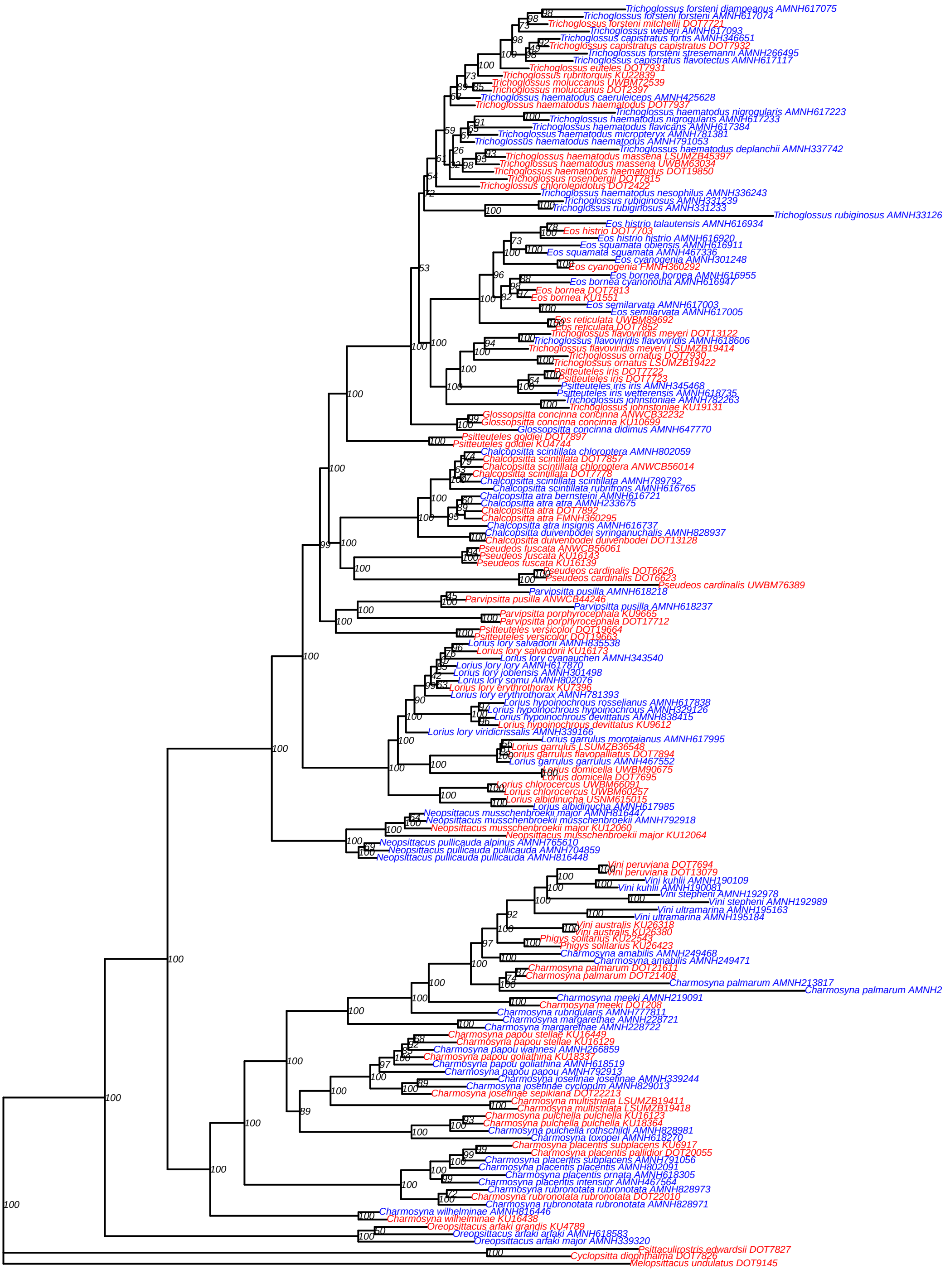

Fig. S6B) Low Coverage: Sample Type

Fig. S7: Best-fit model versus Δ score

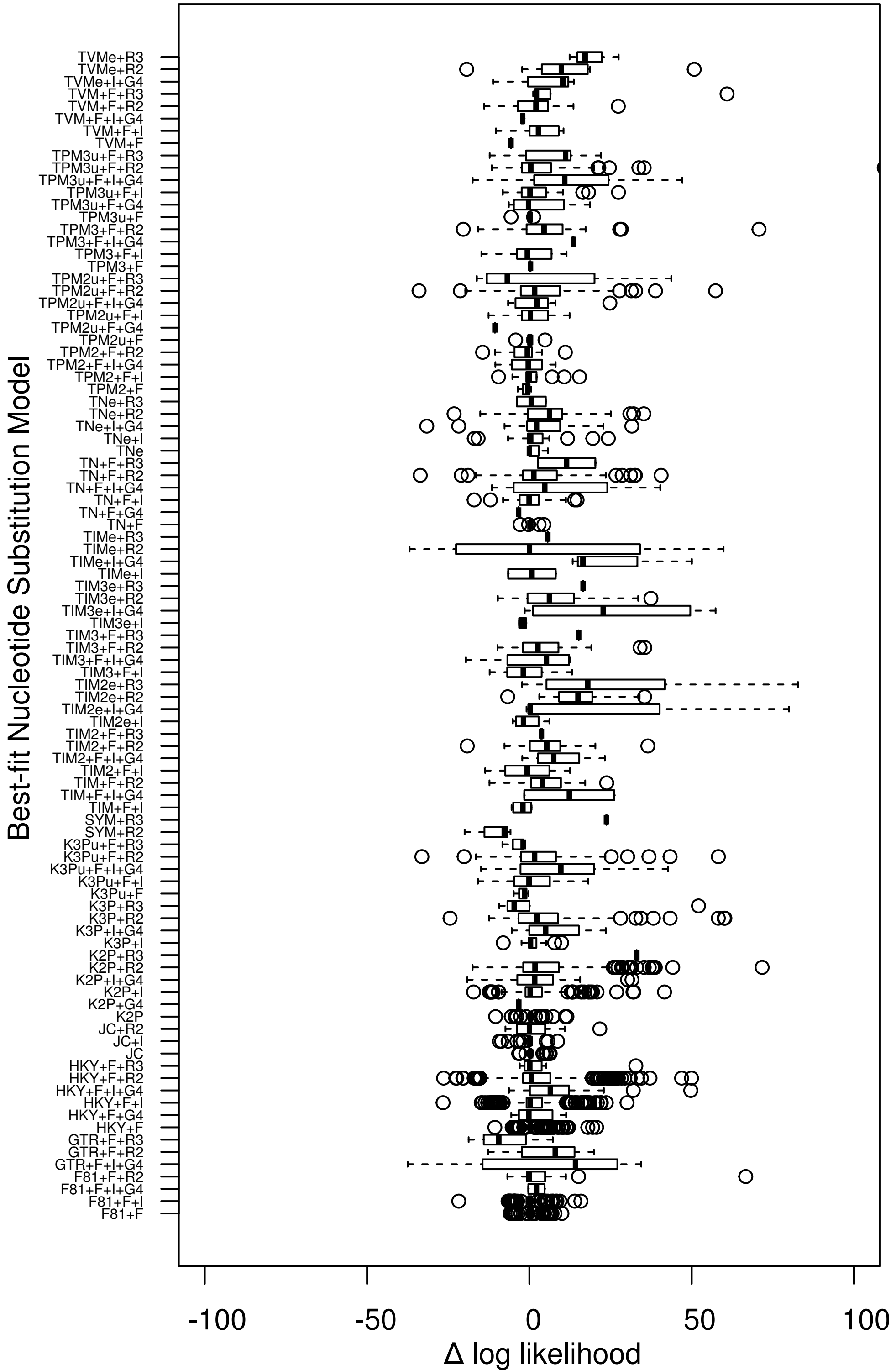

Fig. S8: Likelihood plots forFiltered subclades

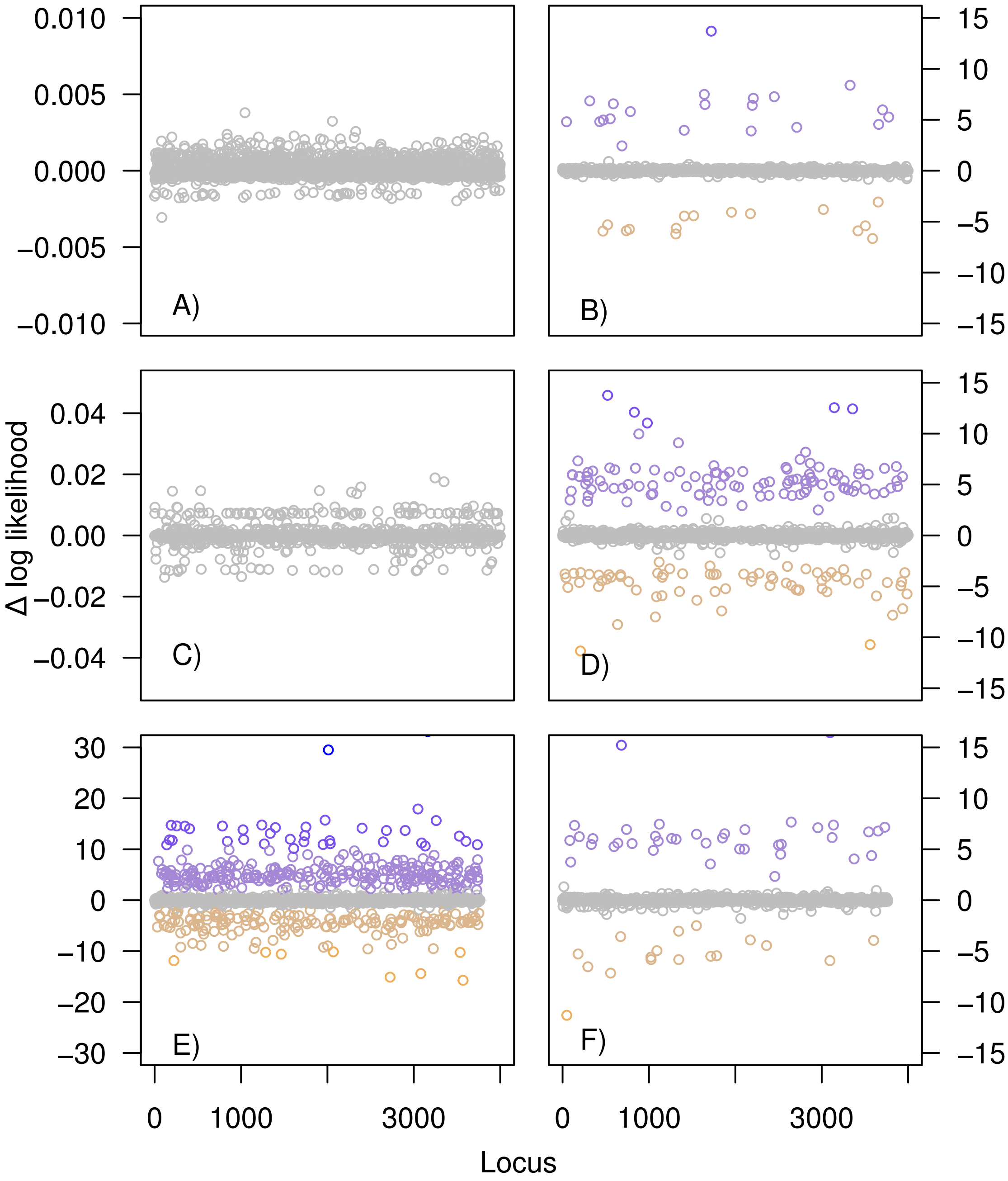

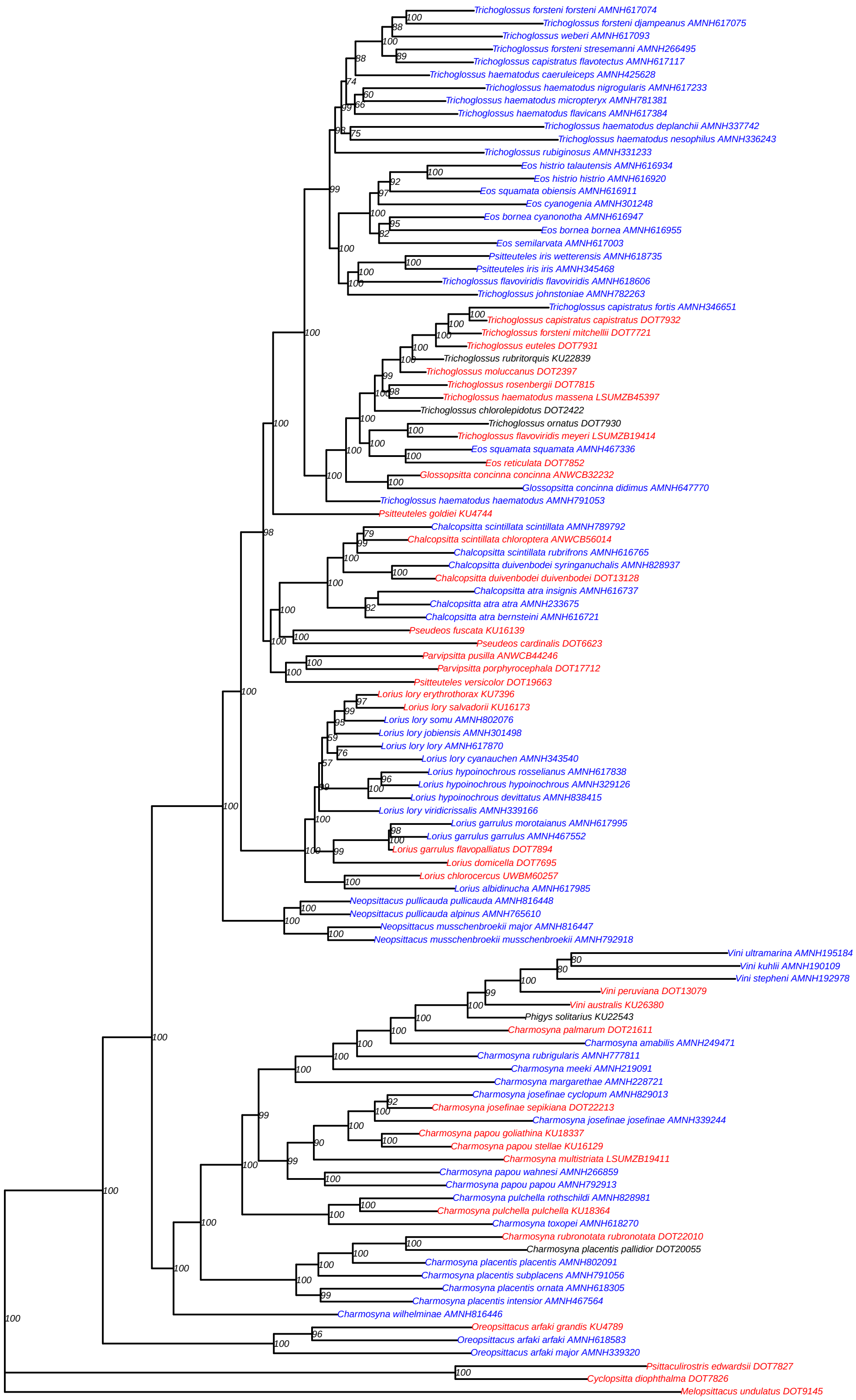

Fig. S9A) Low Coverage: All Sites

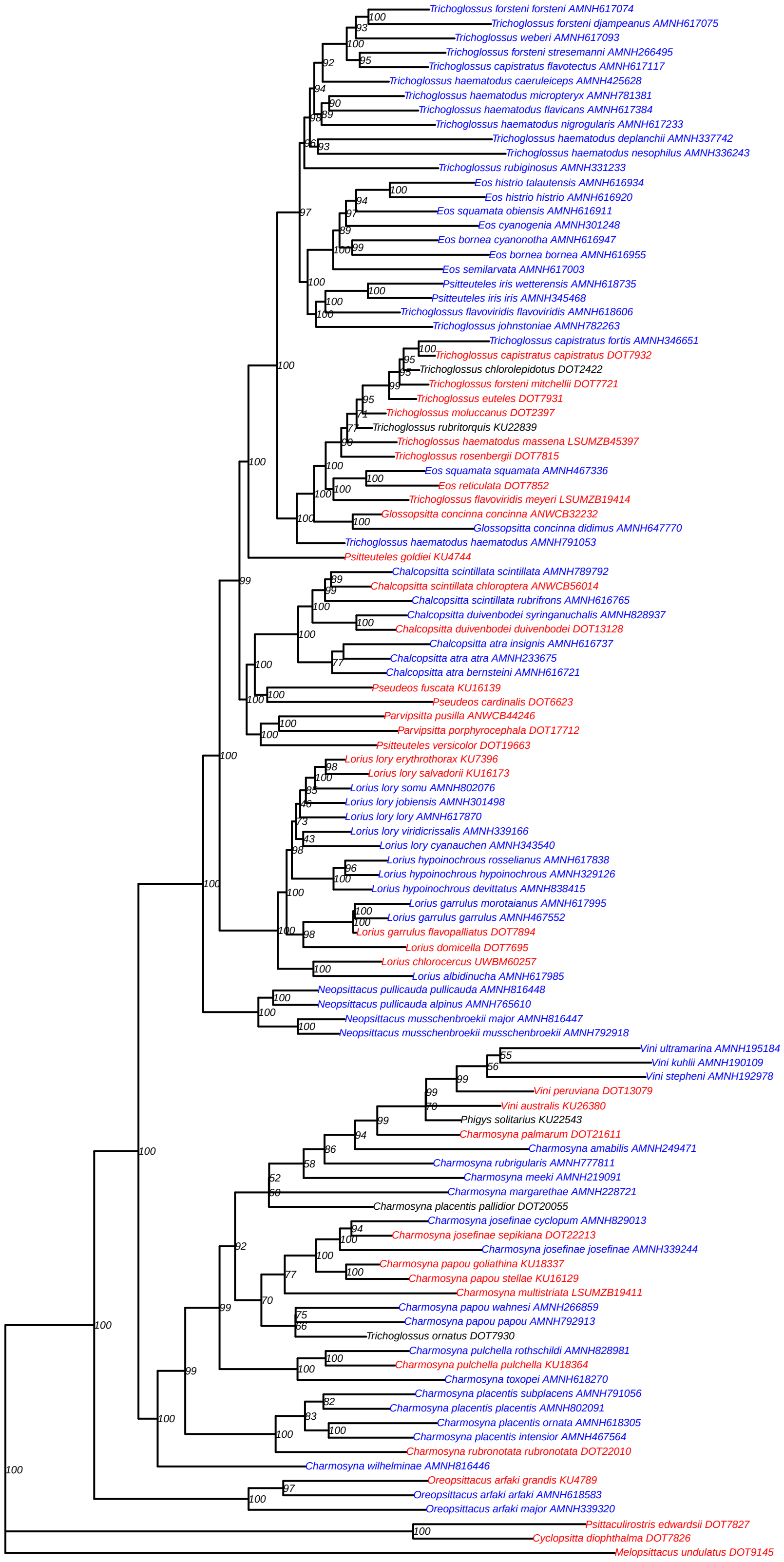

Fig. S9B) Low Coverage: 99.9% PIS Dropped1

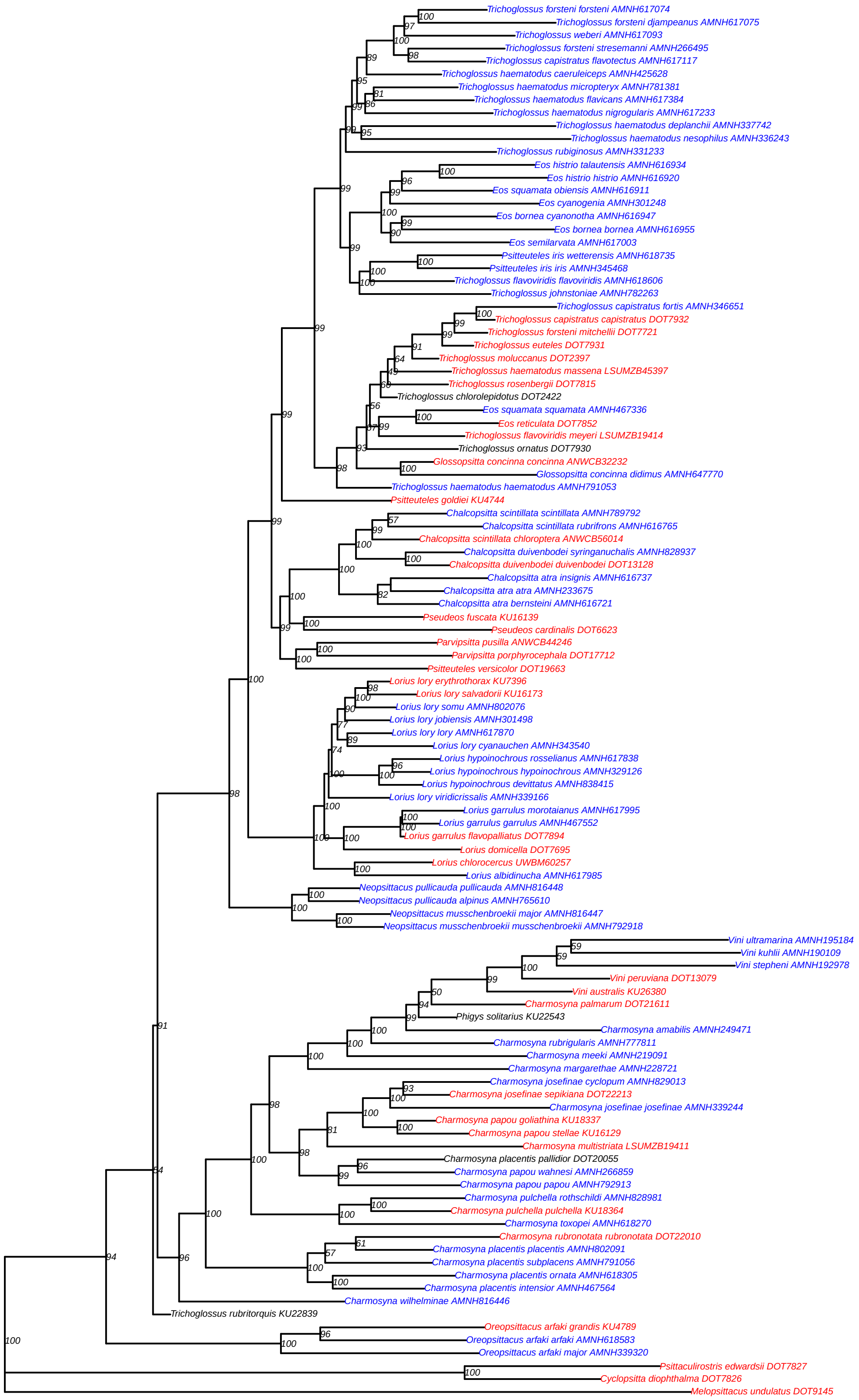

Fig. S9C) Low Coverage: 99.9% PIS Dropped2

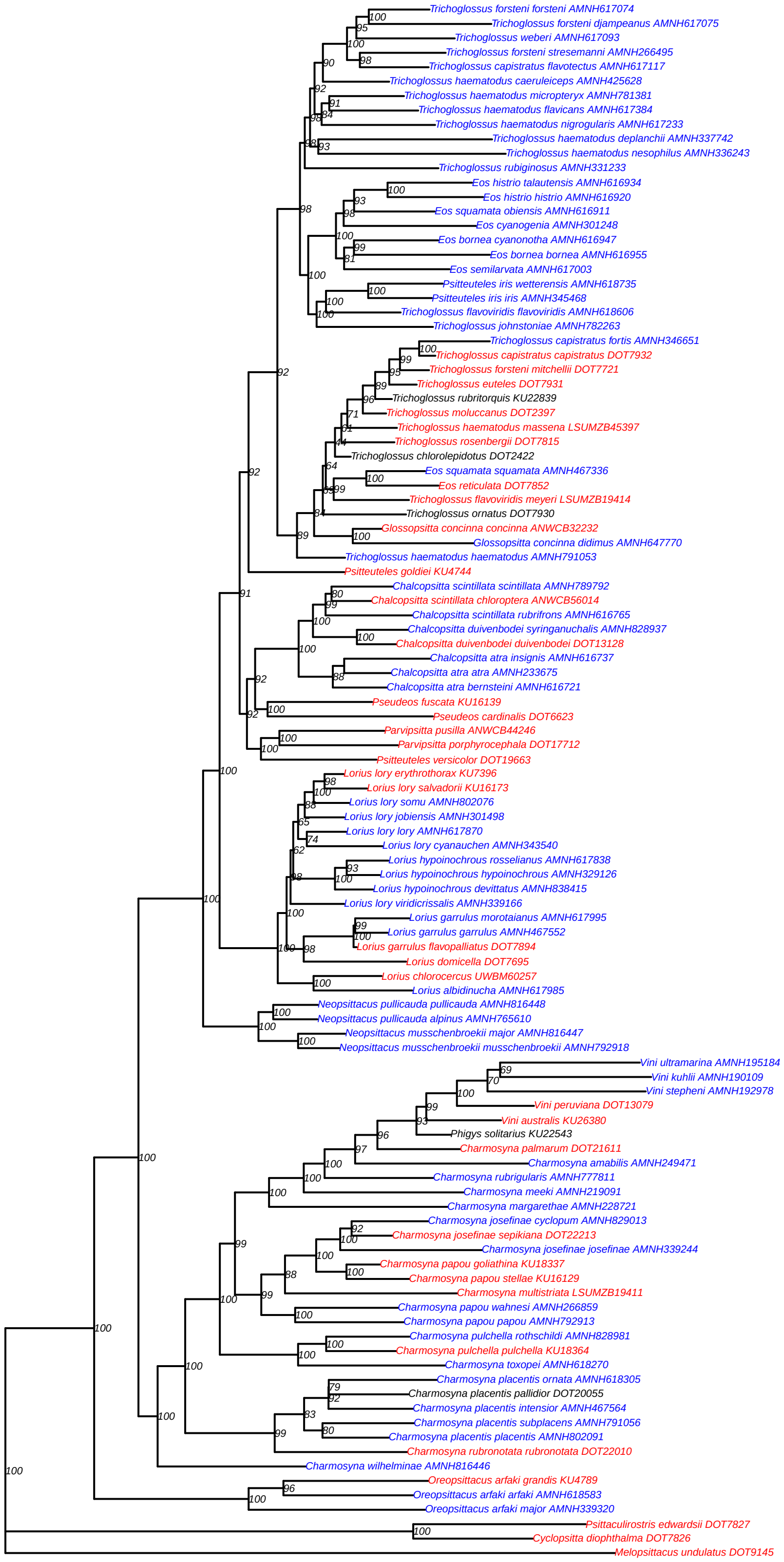

Fig. S9D) Low Coverage: 99.9% PIS Dropped3

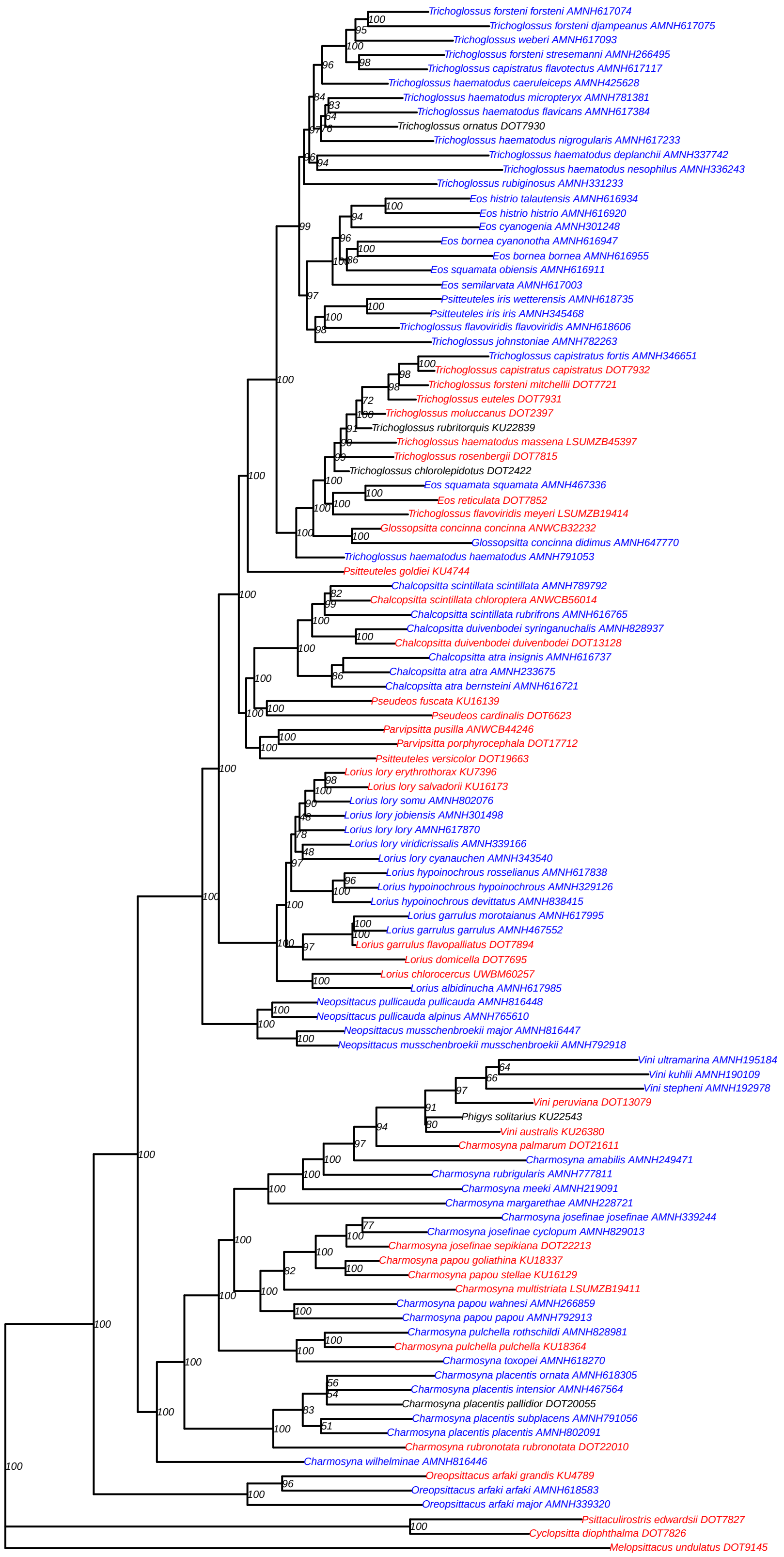

Fig. S9E) Low Coverage: 99.9% PIS Dropped4

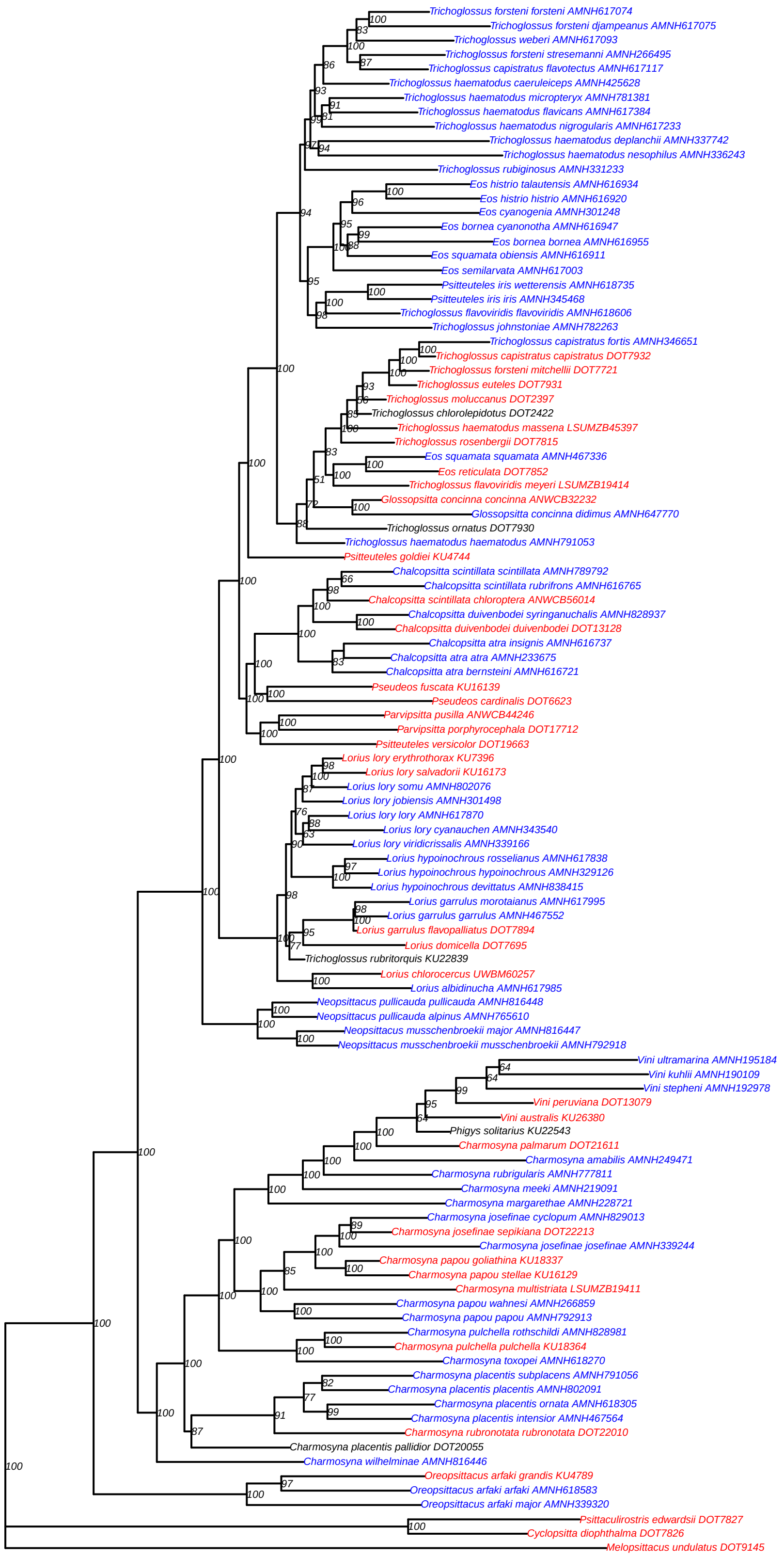

Fig. S9F) Low Coverage: 99.9% PIS Dropped5

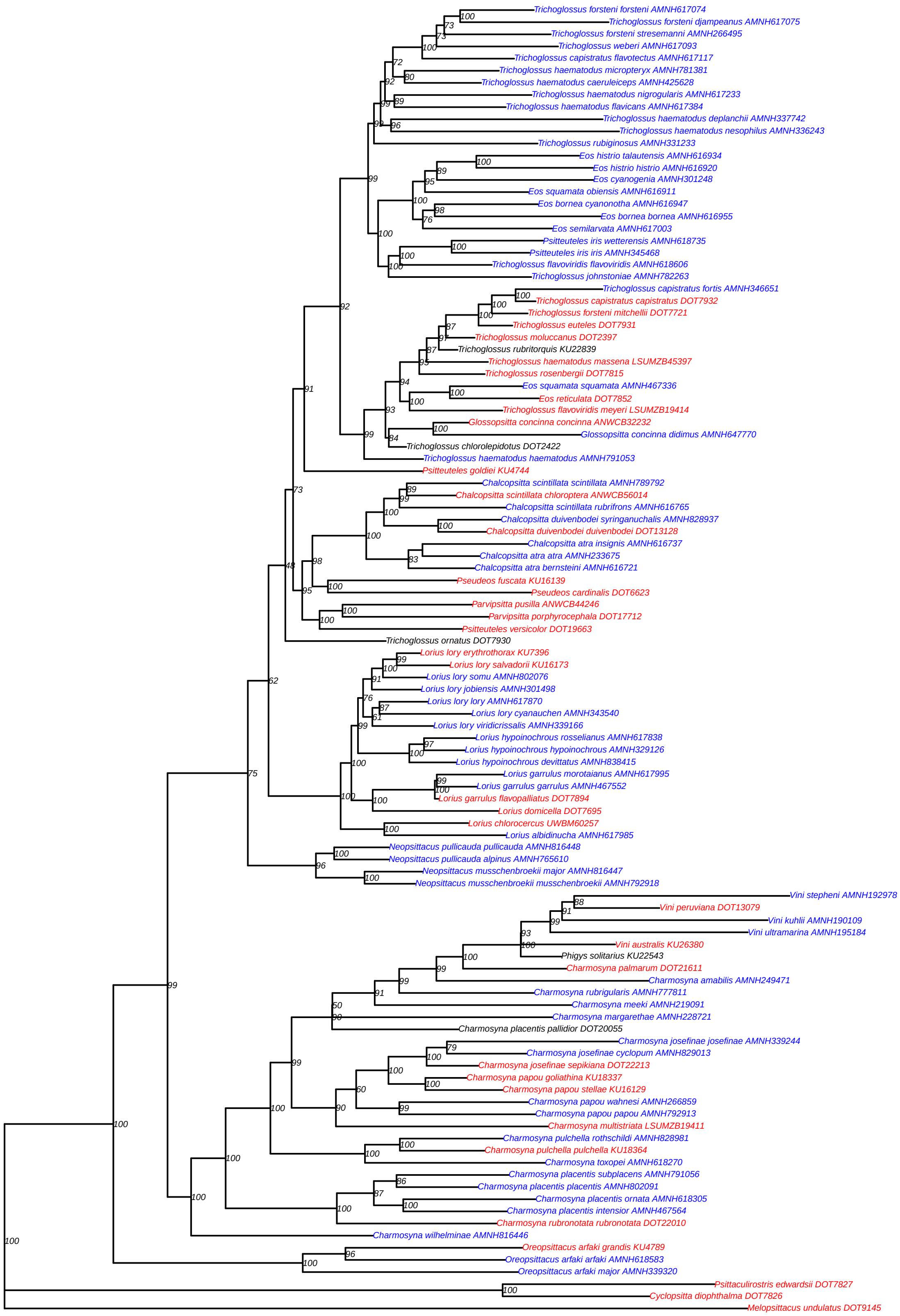

Fig. S9G) Low Coverage: 99.9% PIS Dropped6

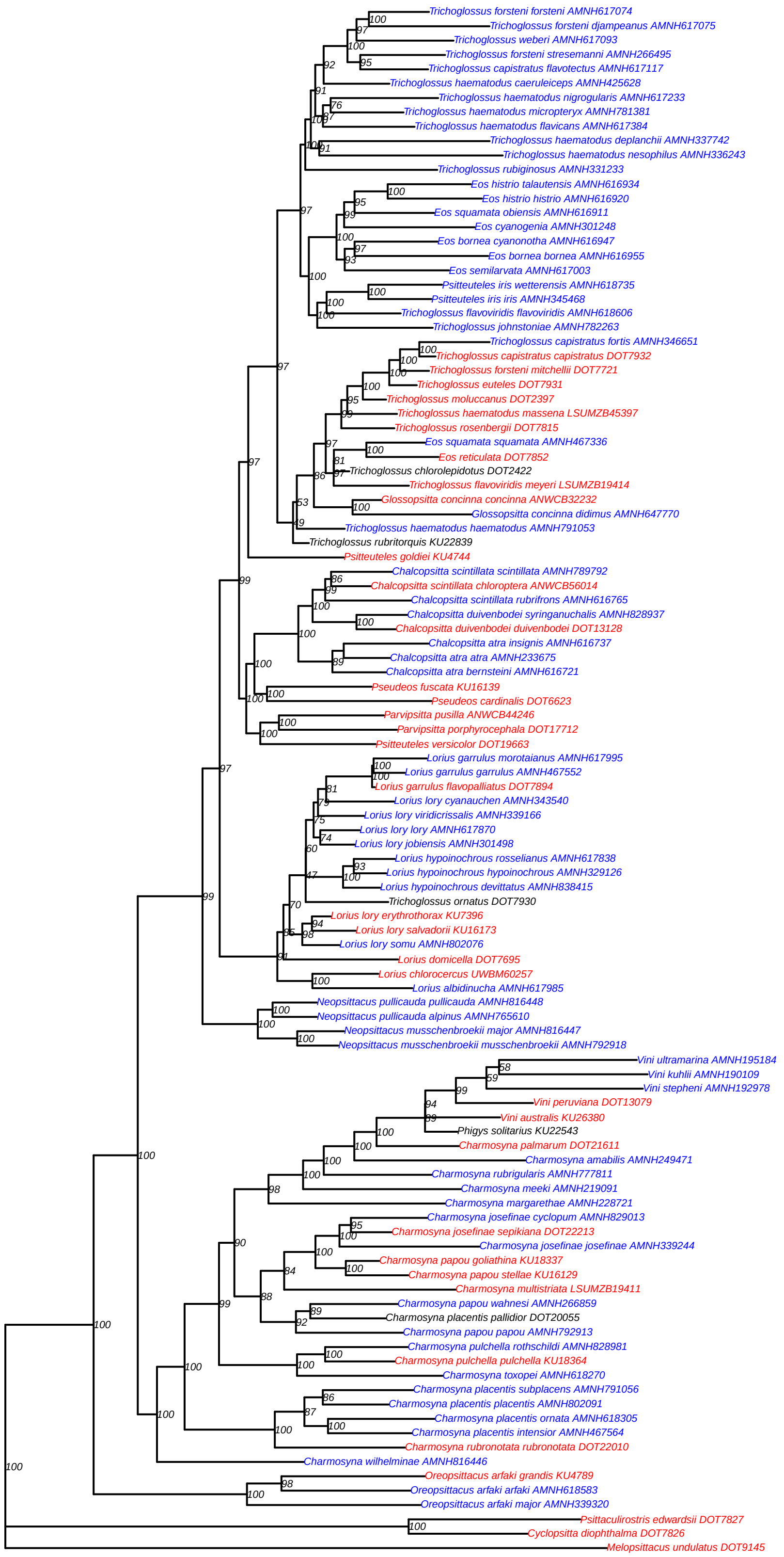

Fig. S9H) Low Coverage: 99.9% PIS Dropped7

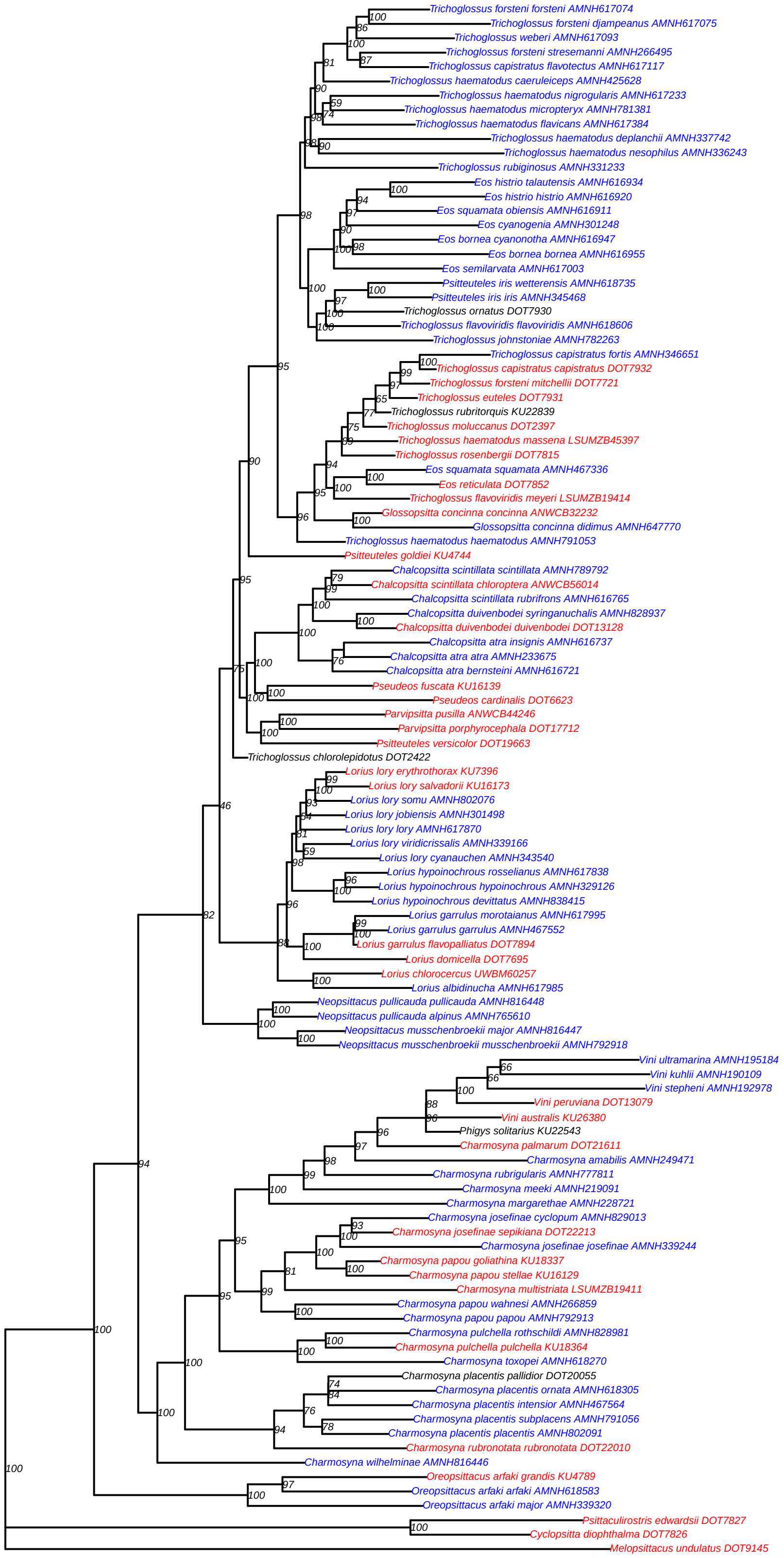

Fig. S9l) Low Coverage: 99.9% PIS Dropped8

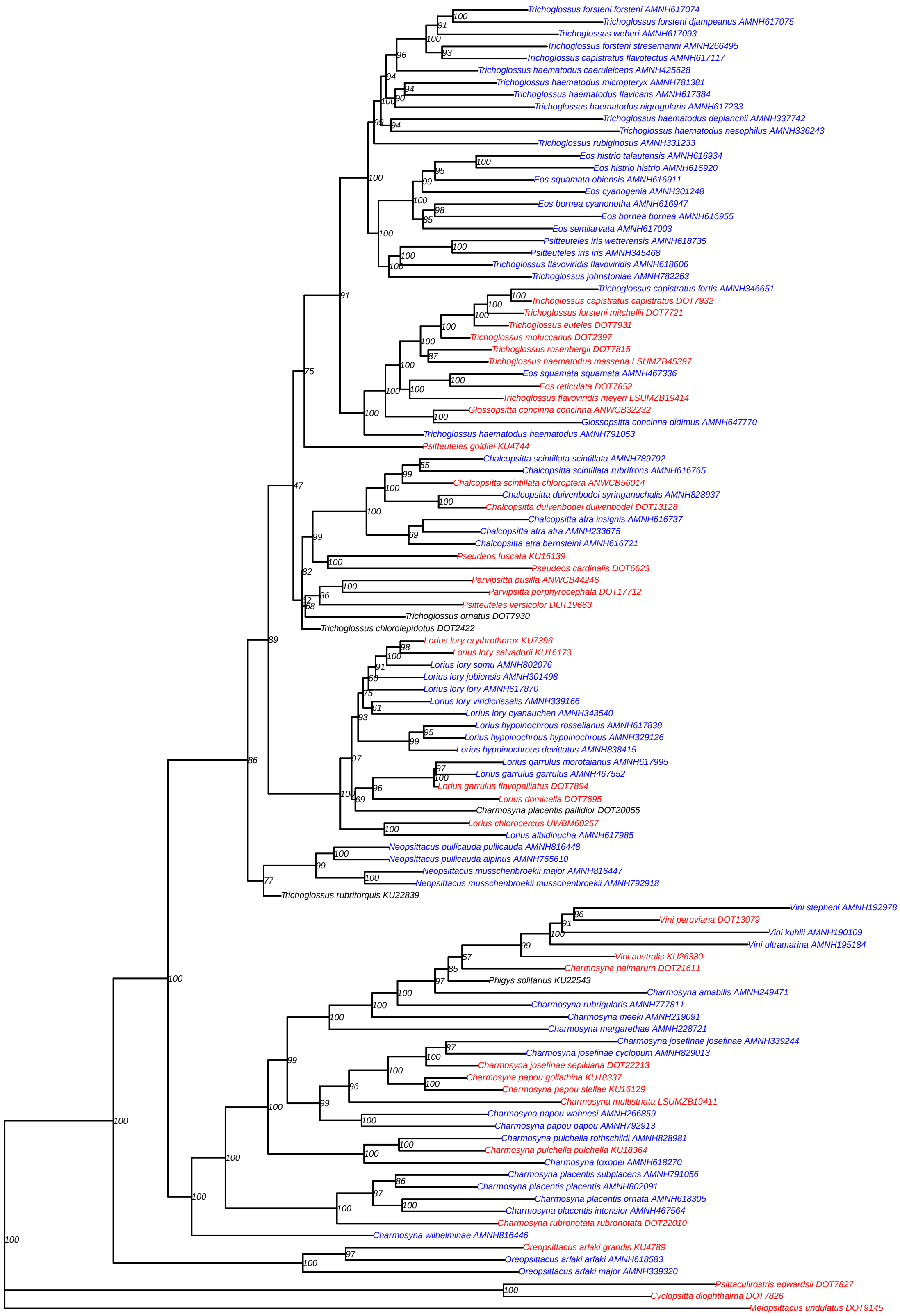

Fig. S9J) Low Coverage: 99.9% PIS Dropped9

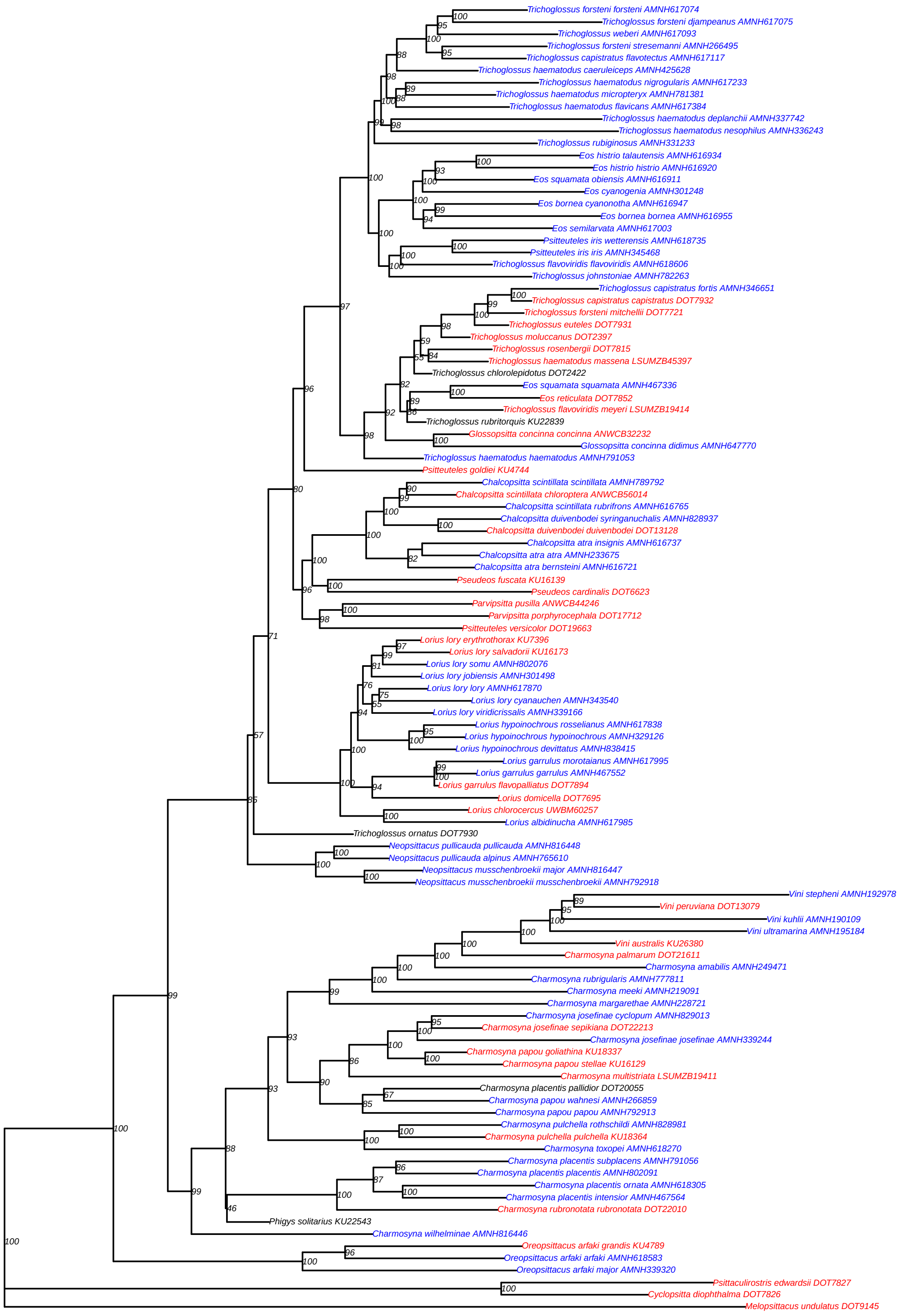

Fig. S9K) Low Coverage: 99.9% PIS Dropped10

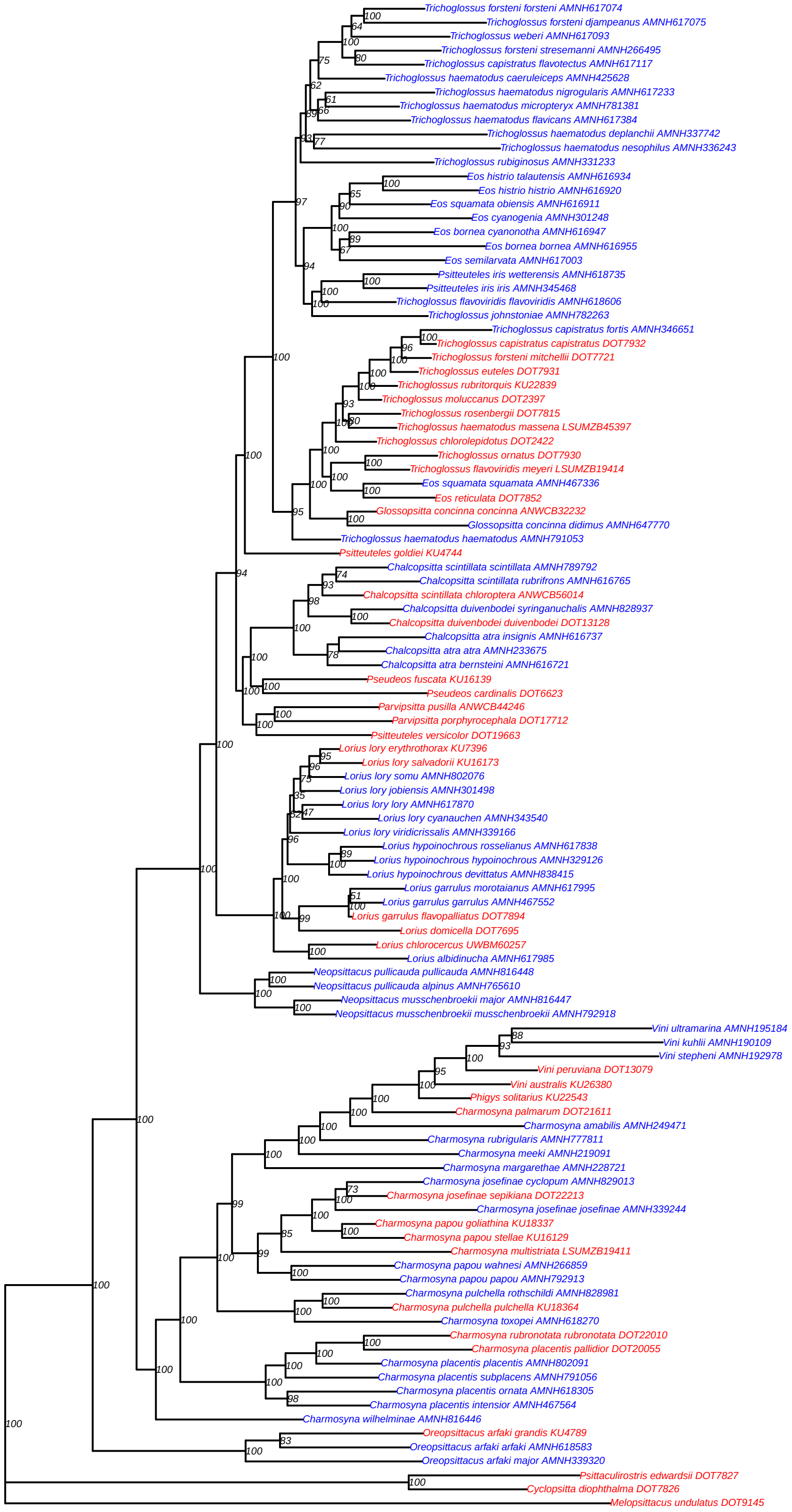

Fig. S10A) Low Coverage: All Sites

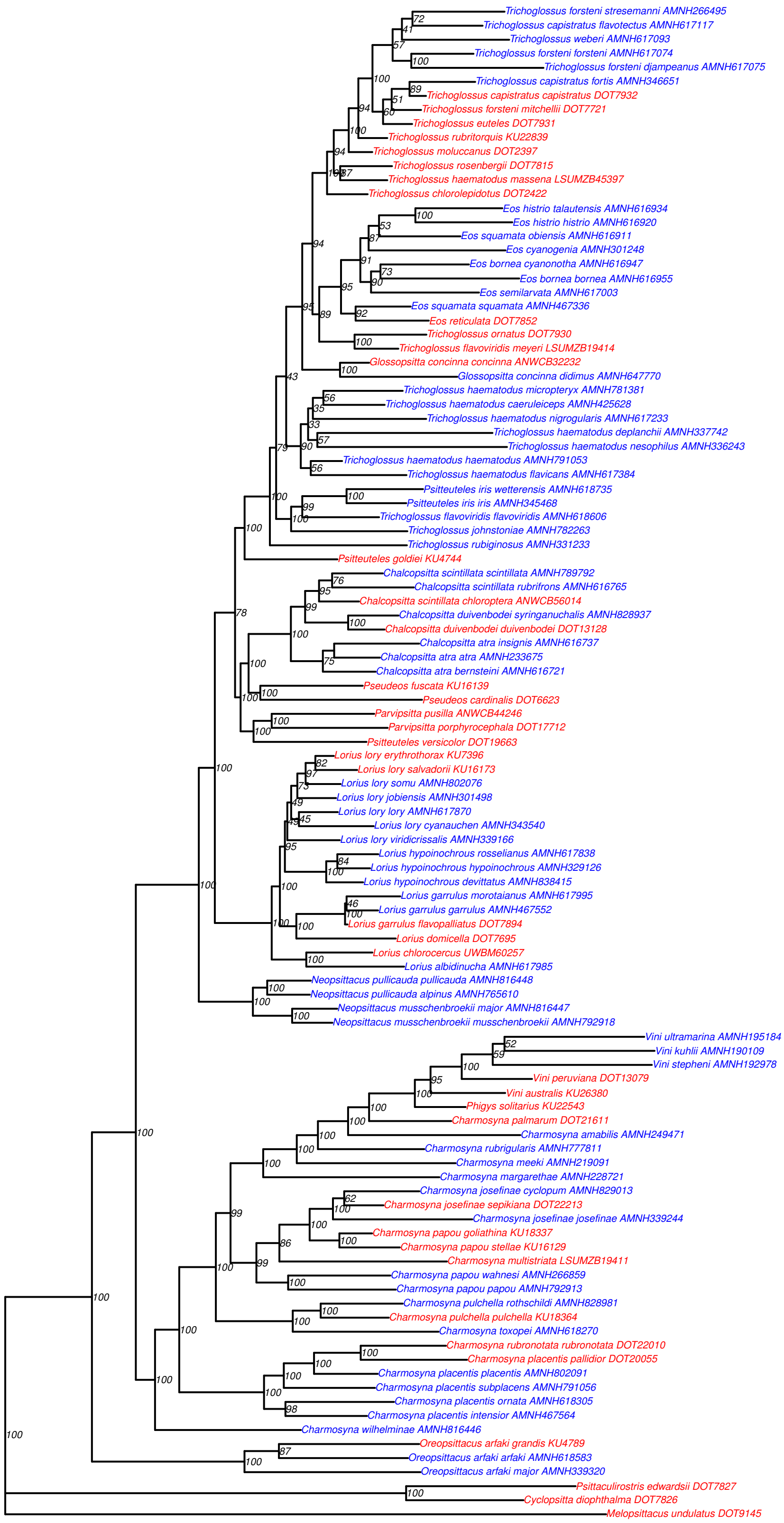

Fig. S10B) Low Coverage: Δ s–lk > 20 Excluded

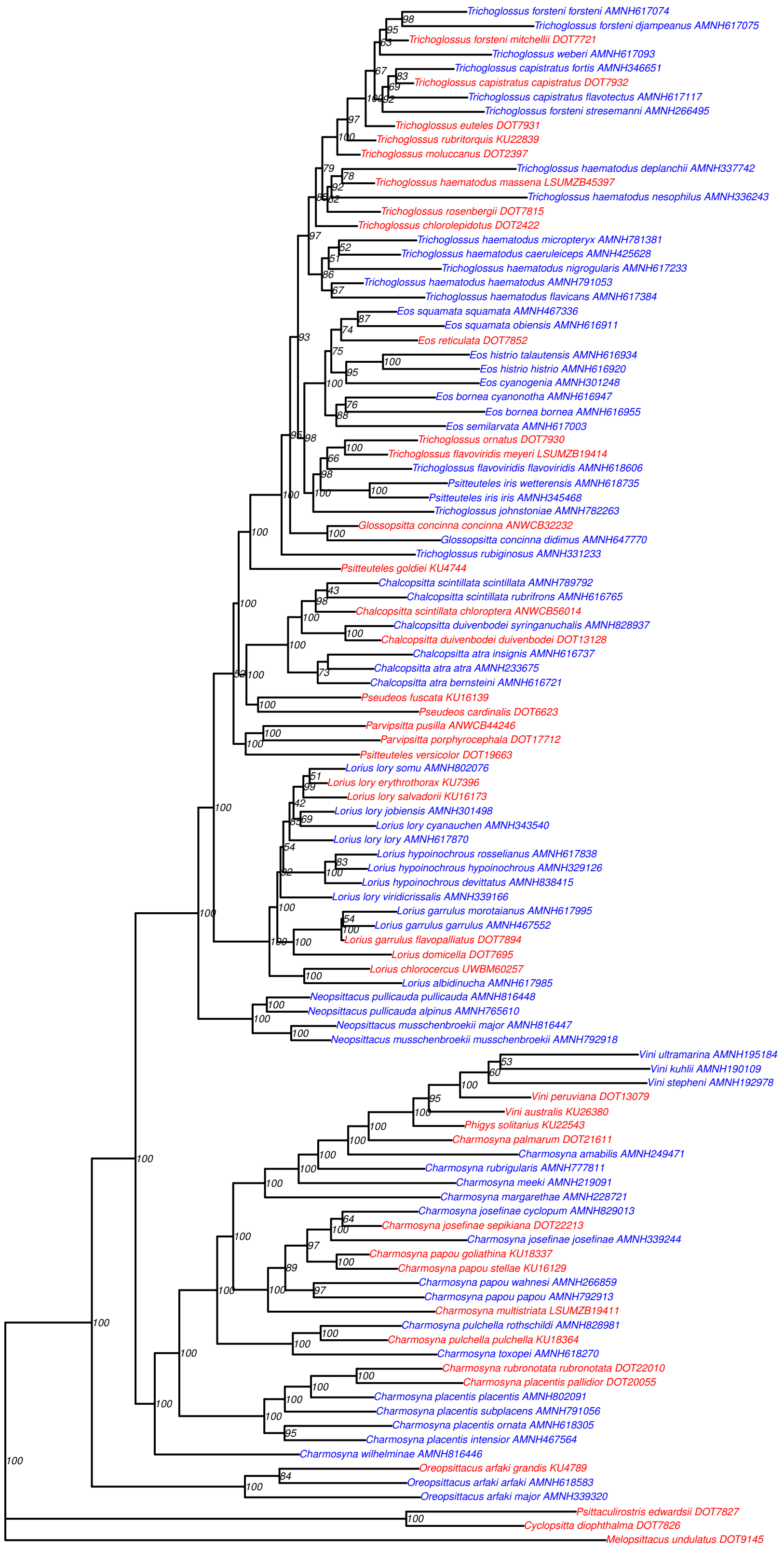

Fig. S10C) Low Coverage: Δ s–lk > 10 Excluded

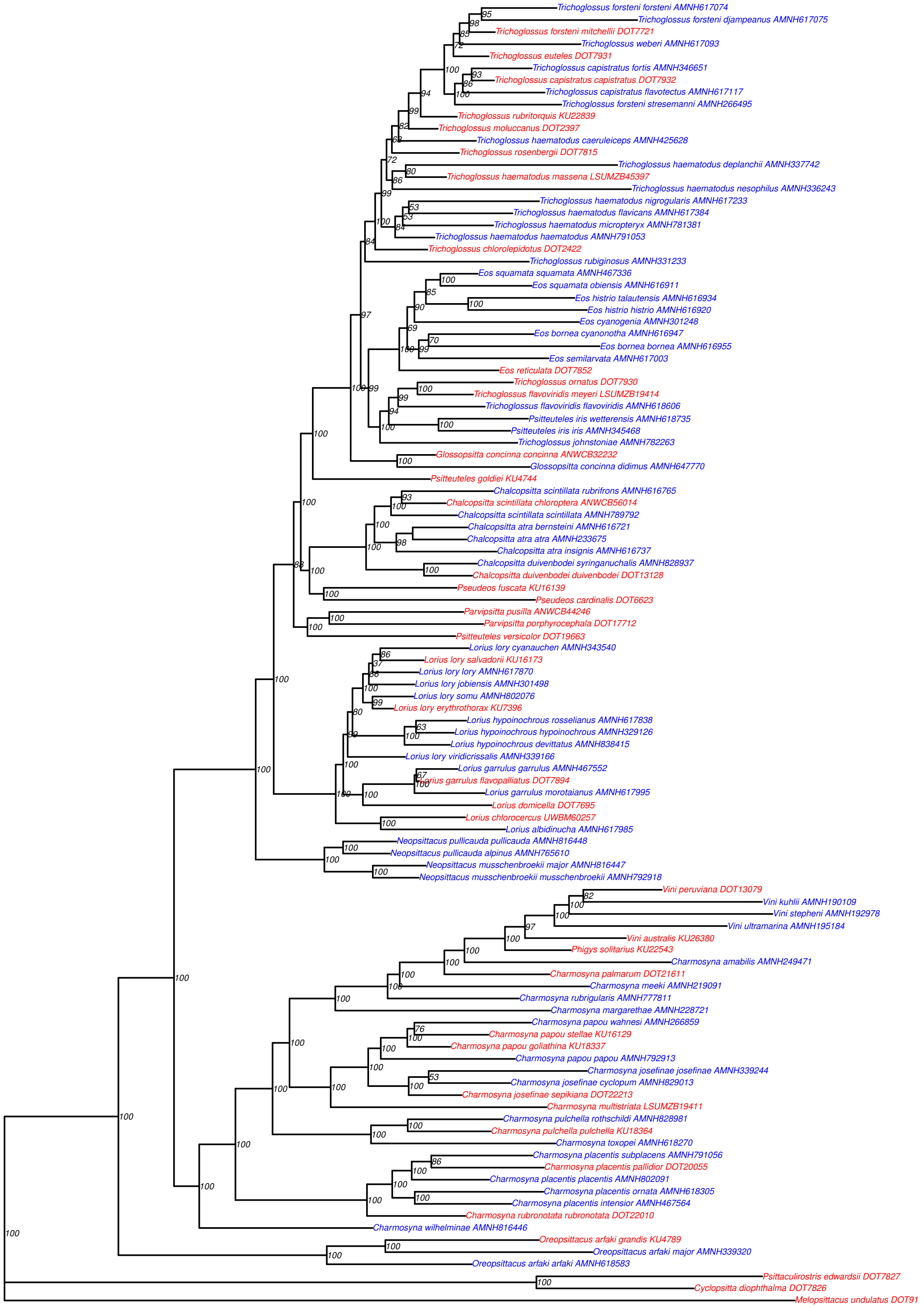

Fig. S10D) Low Coverage: Δ s–lk > 2 Excluded

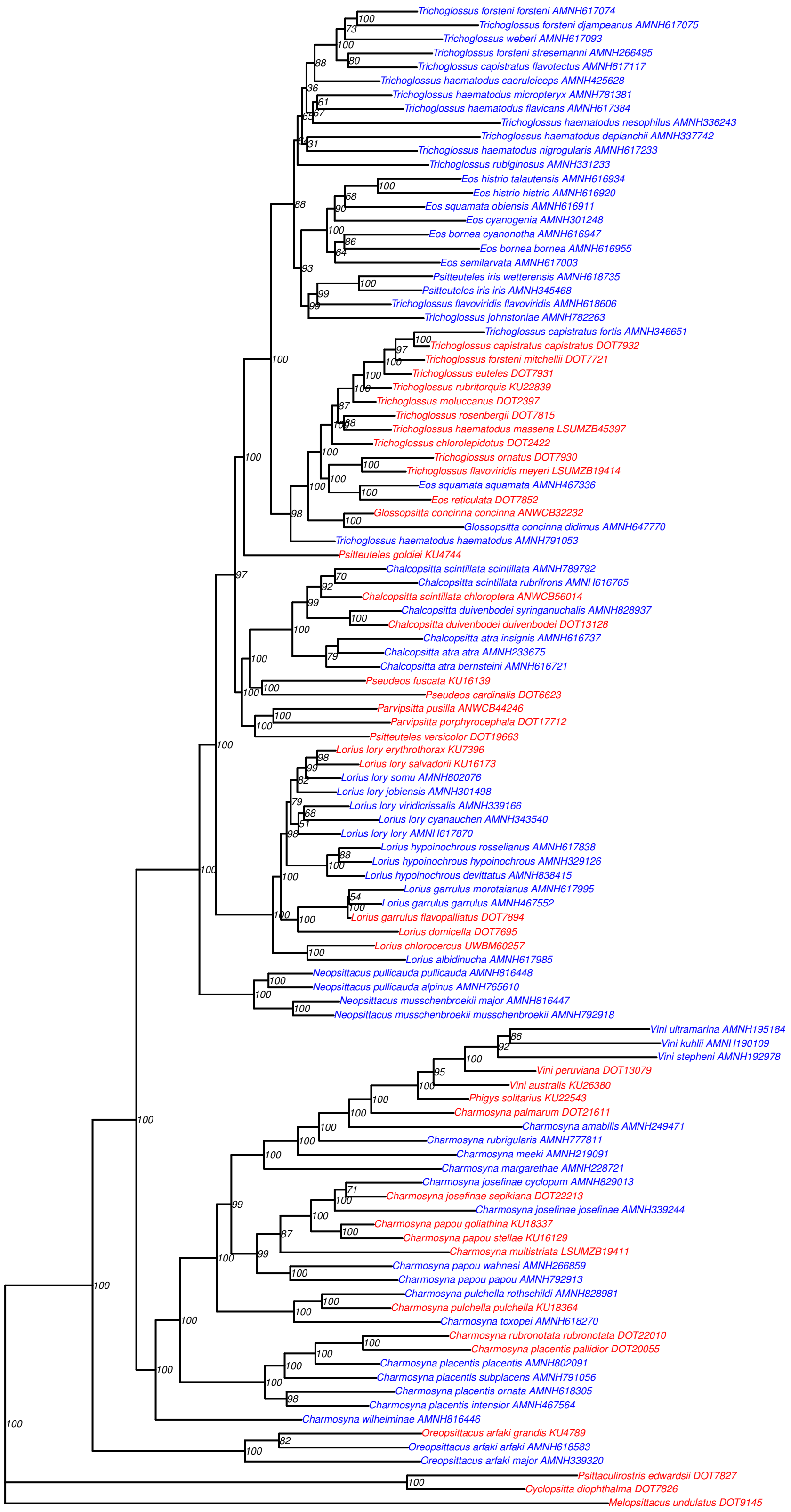

Fig. S10E) Low Coverage: Δ s–lk < –20 Excluded

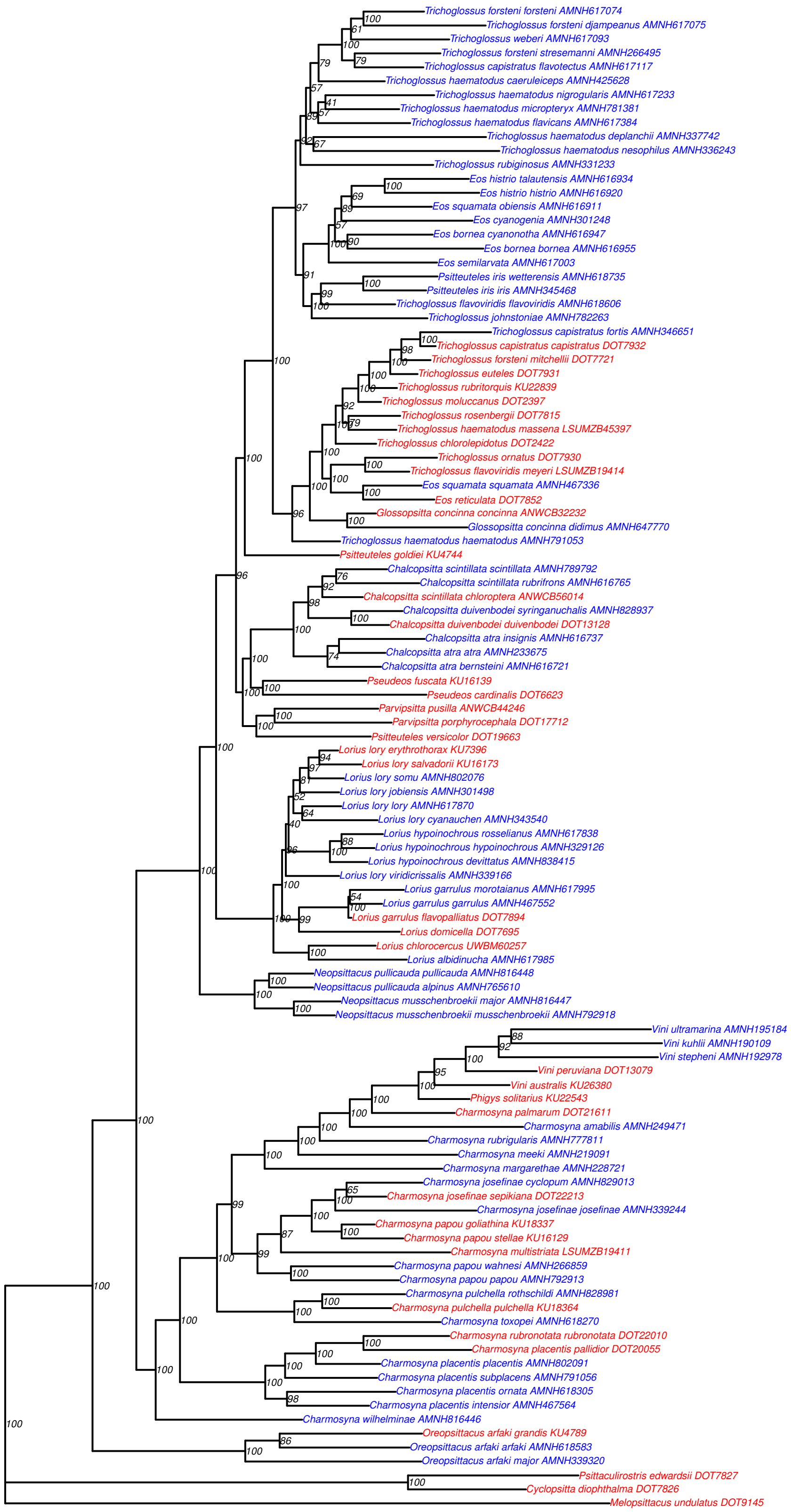

Fig. S10F) Low Coverage: Δ s–lk < –10 Excluded

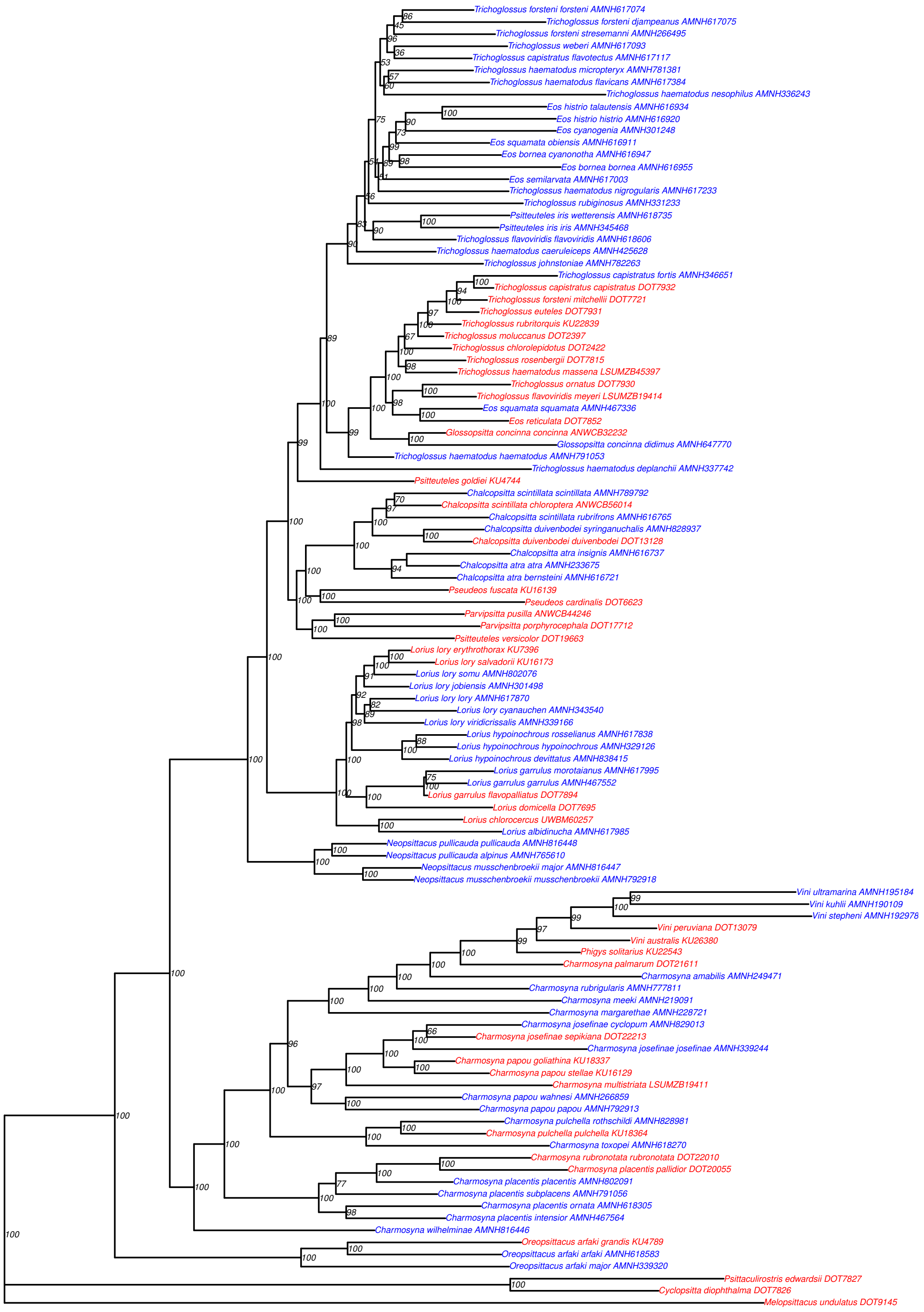

Fig. S10G) Low Coverage: Δ s–lk < –2 Excluded

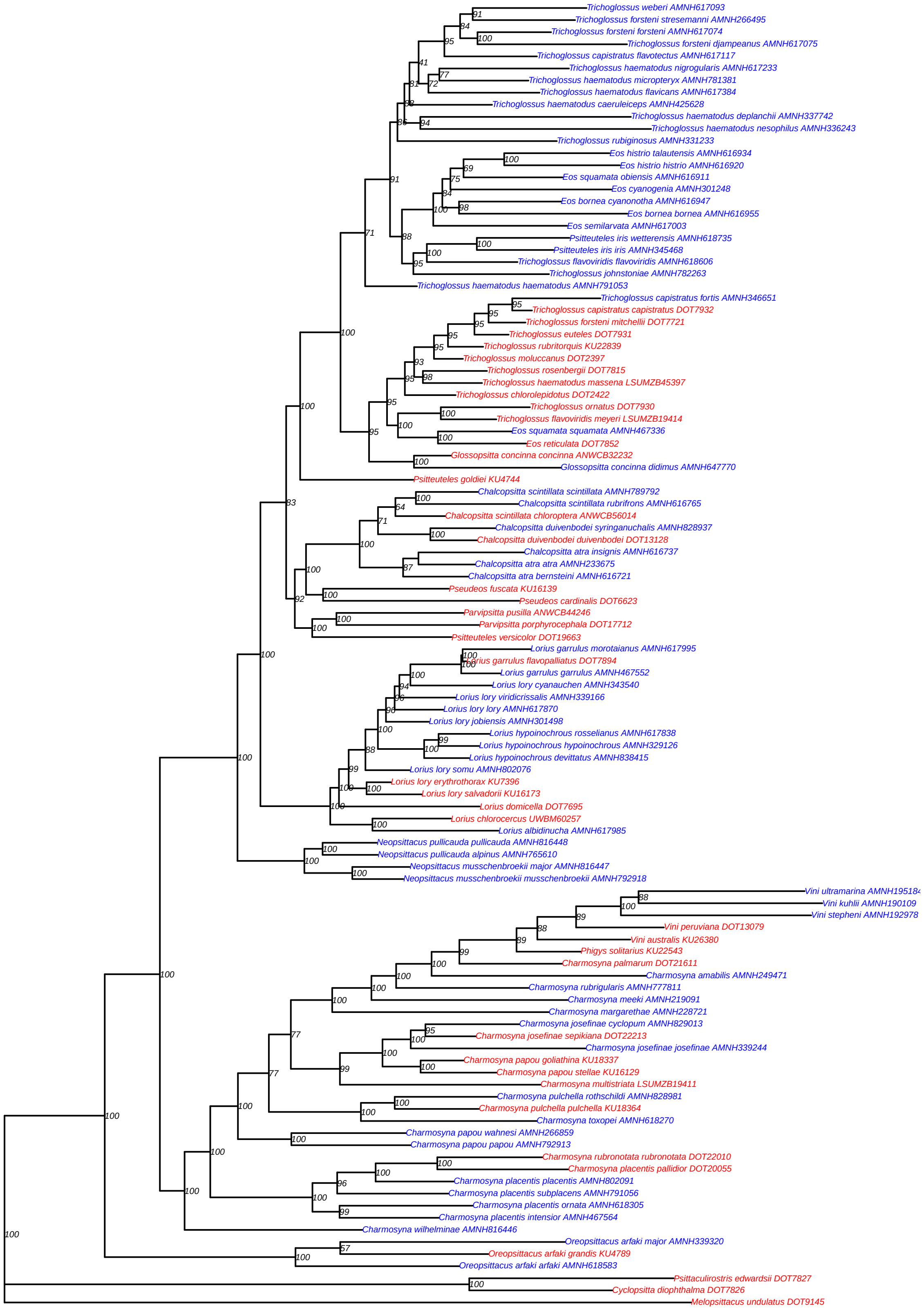

Fig. S11A) Low Coverage: 0% Data Completeness

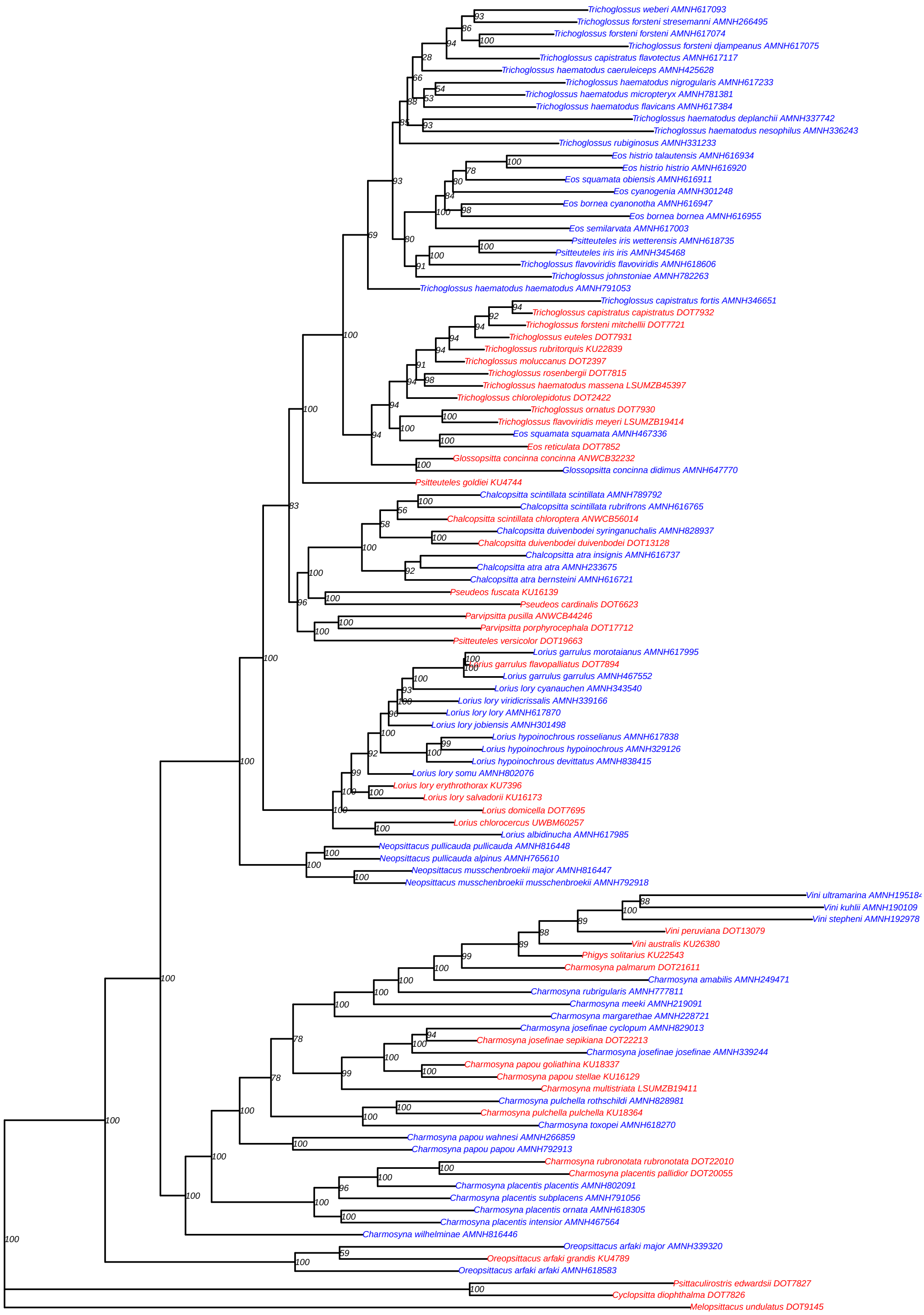

Fig. S11B) Low Coverage: 10% Data Completeness

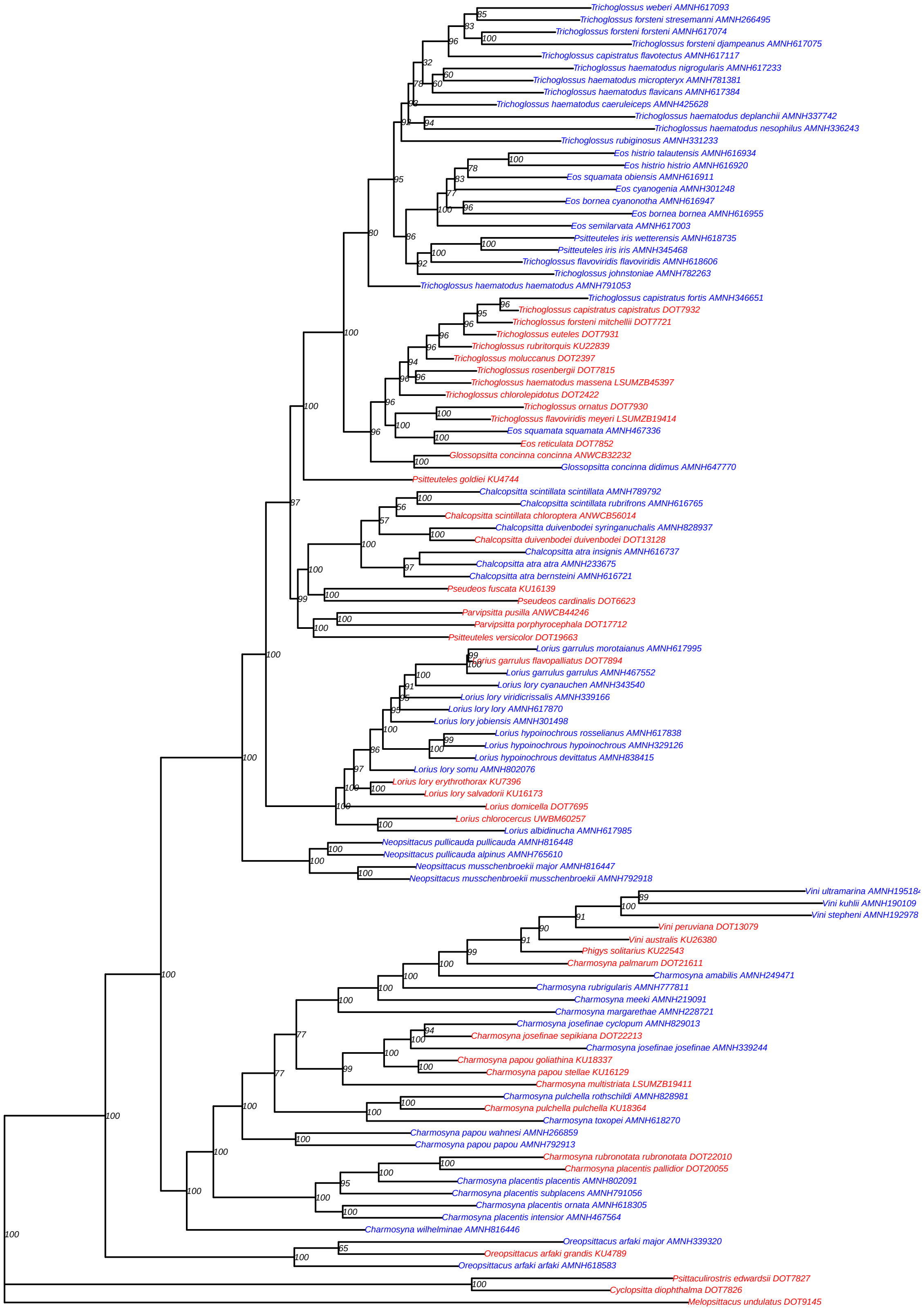

Fig. S11C) Low Coverage: 20% Data Completeness

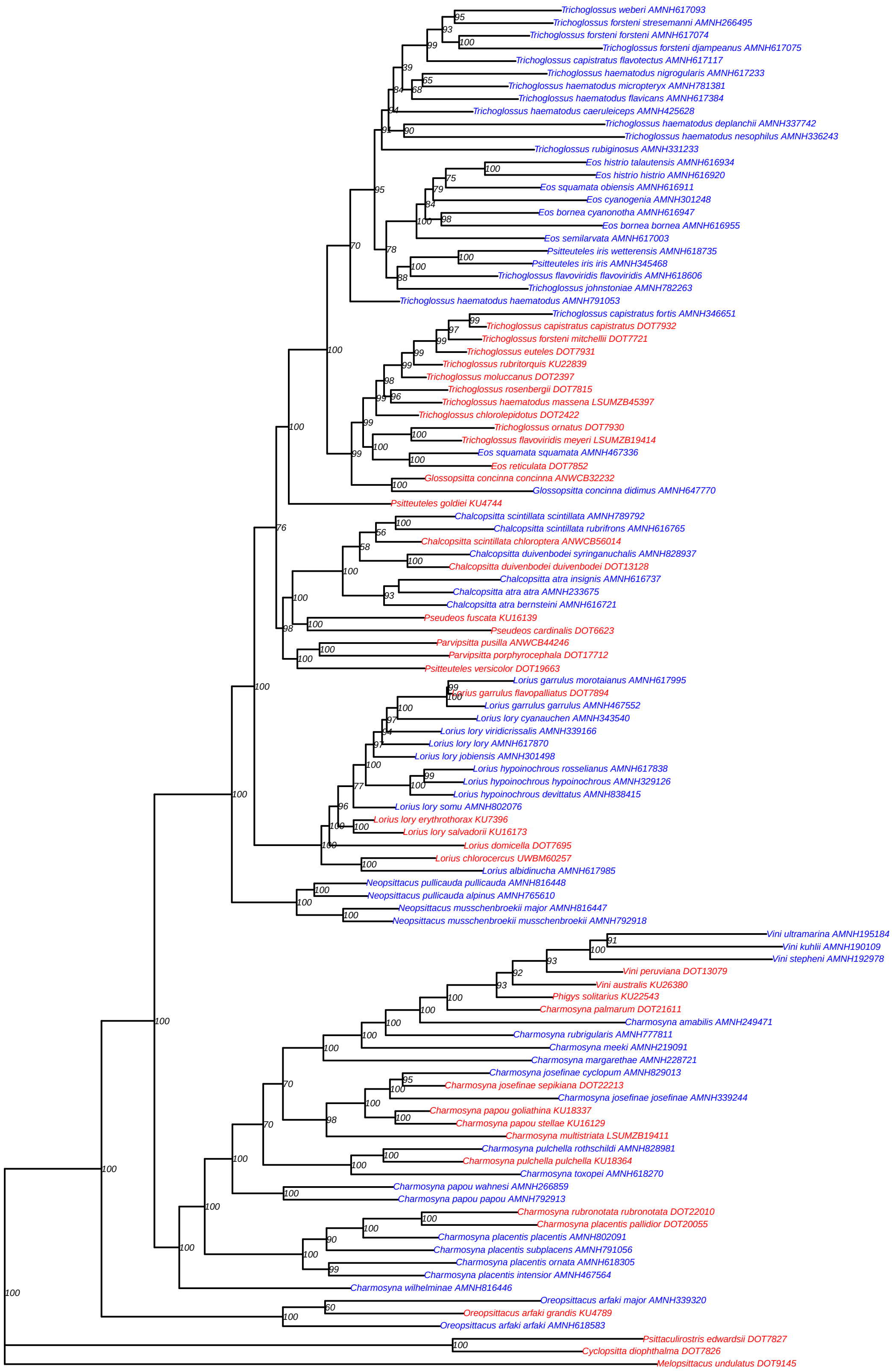

Fig. S11D) Low Coverage: 30% Data Completeness

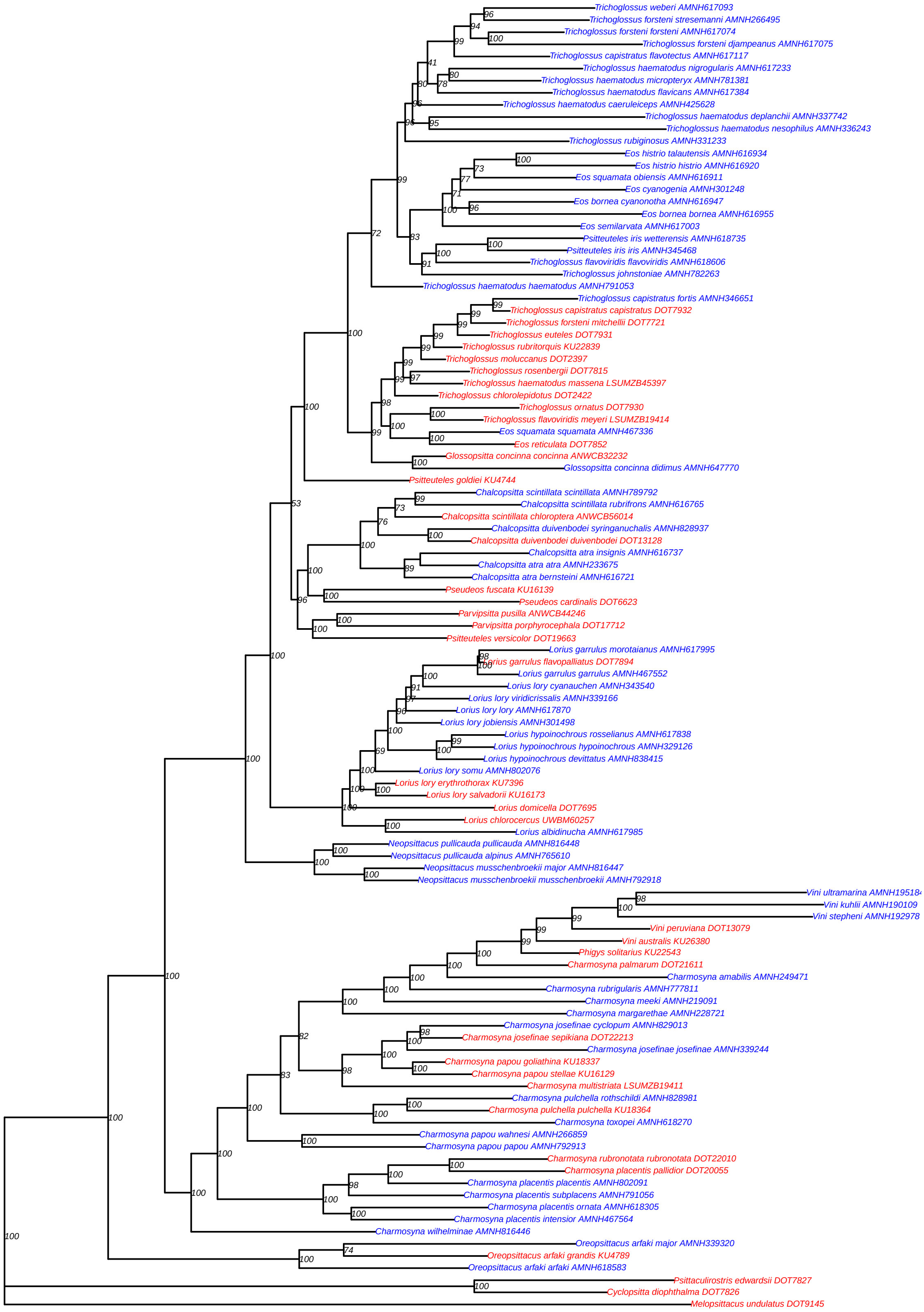

Fig. S11E) Low Coverage: 40% Data Completeness

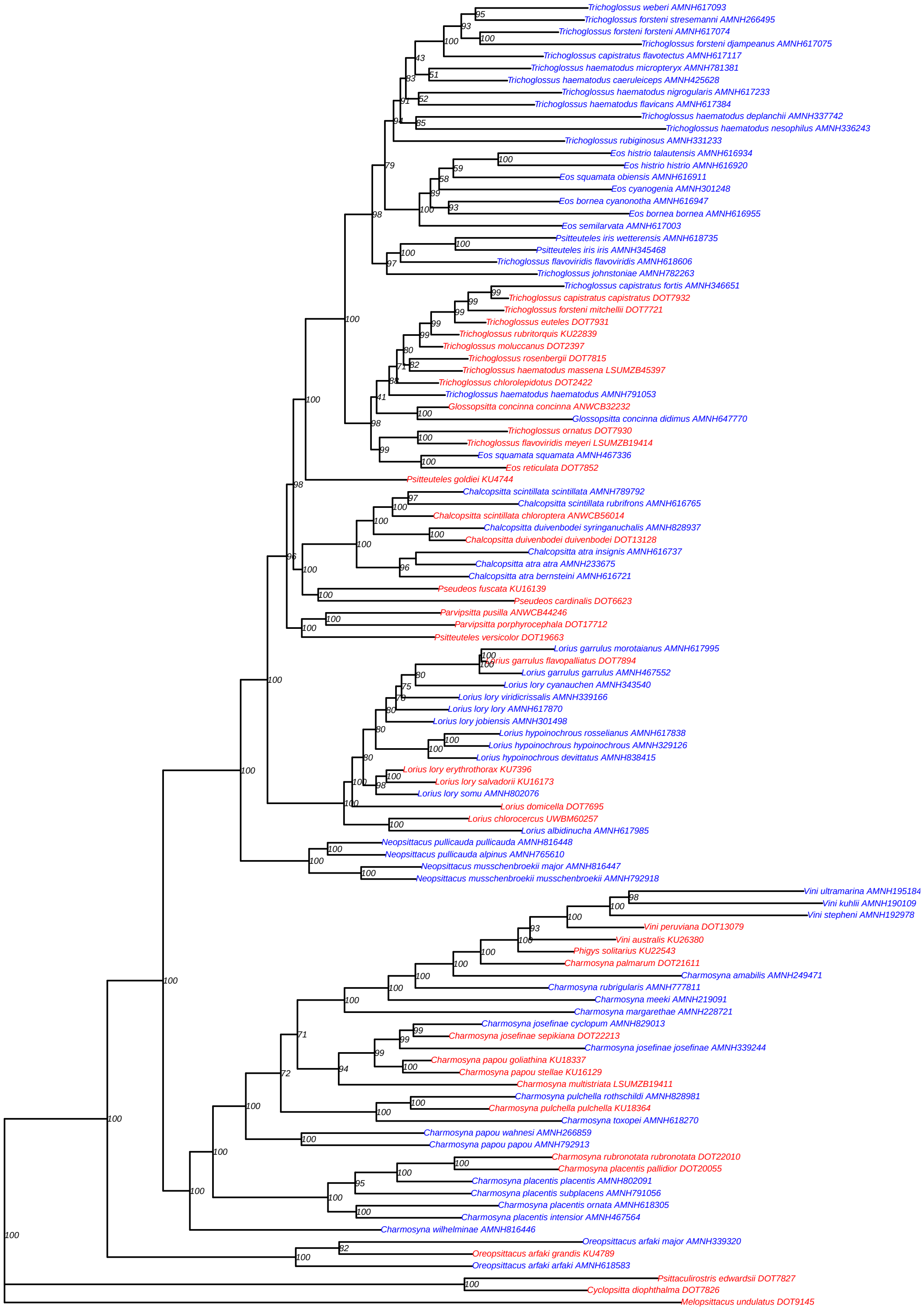

Fig. S11F) Low Coverage: 50% Data Completeness

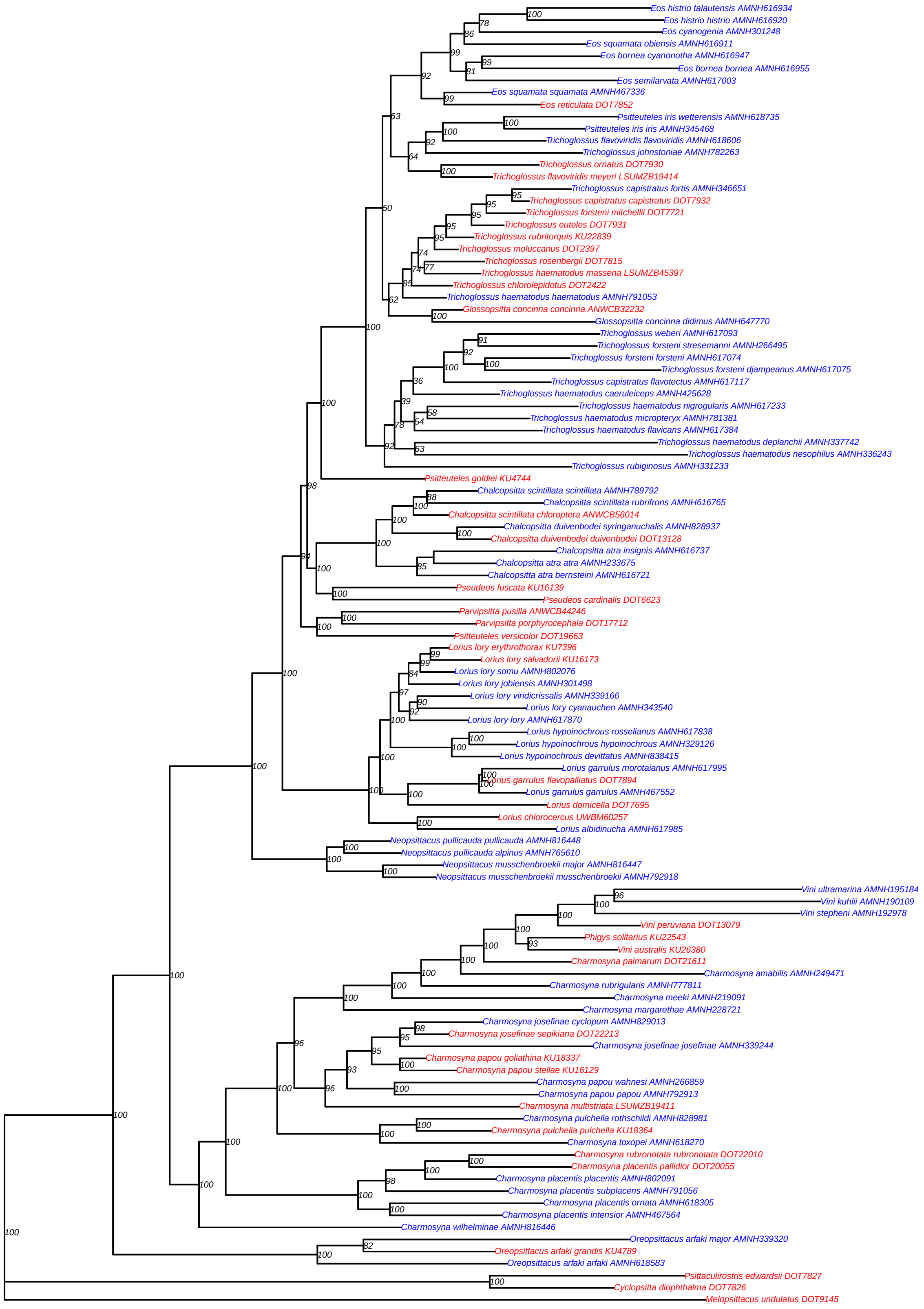

Fig. S11G) Low Coverage: 60% Data Completeness

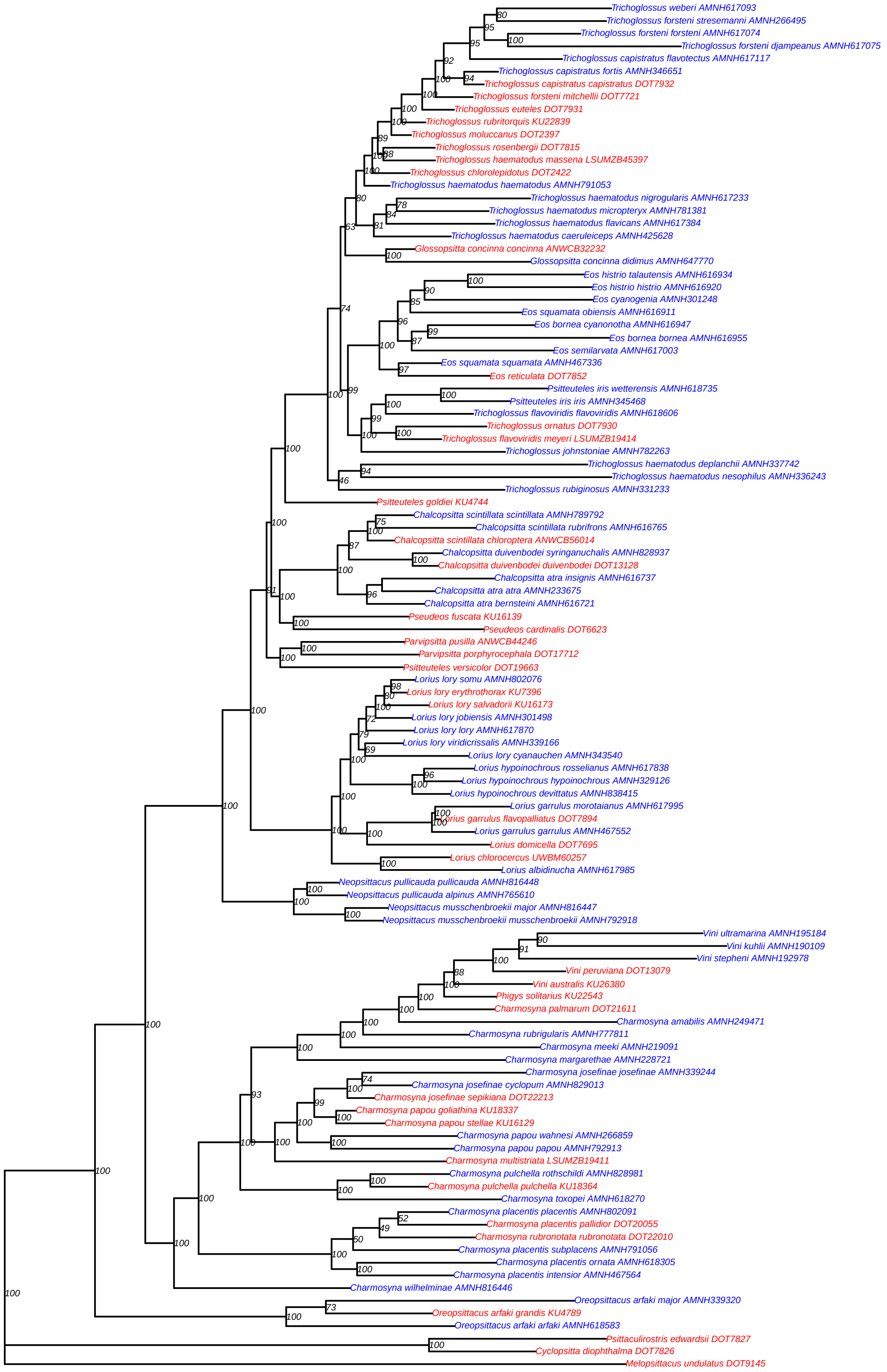

Fig. S11H) Low Coverage: 70% Data Completeness

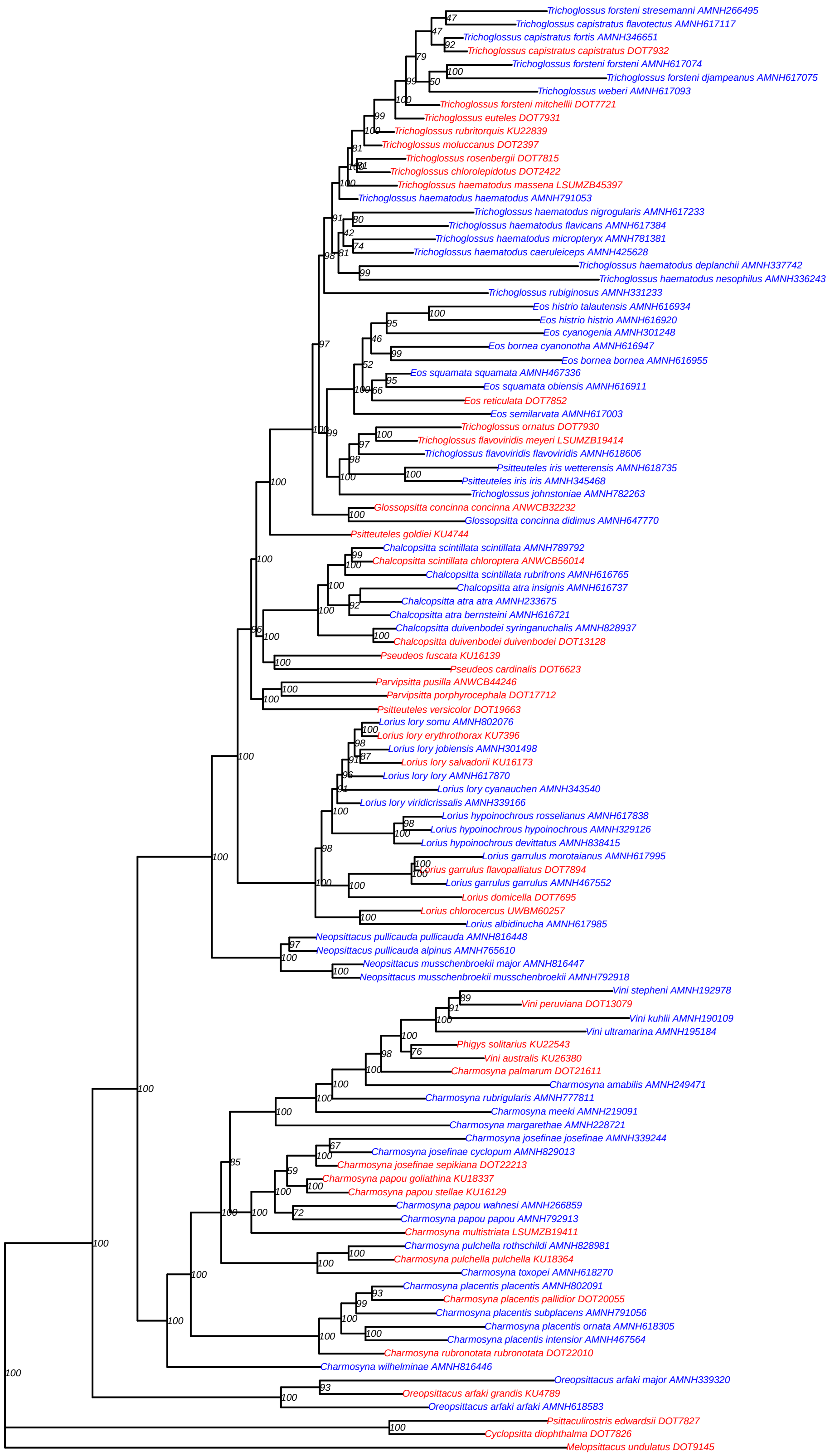

Fig. S11l) Low Coverage: 80% Data Completeness

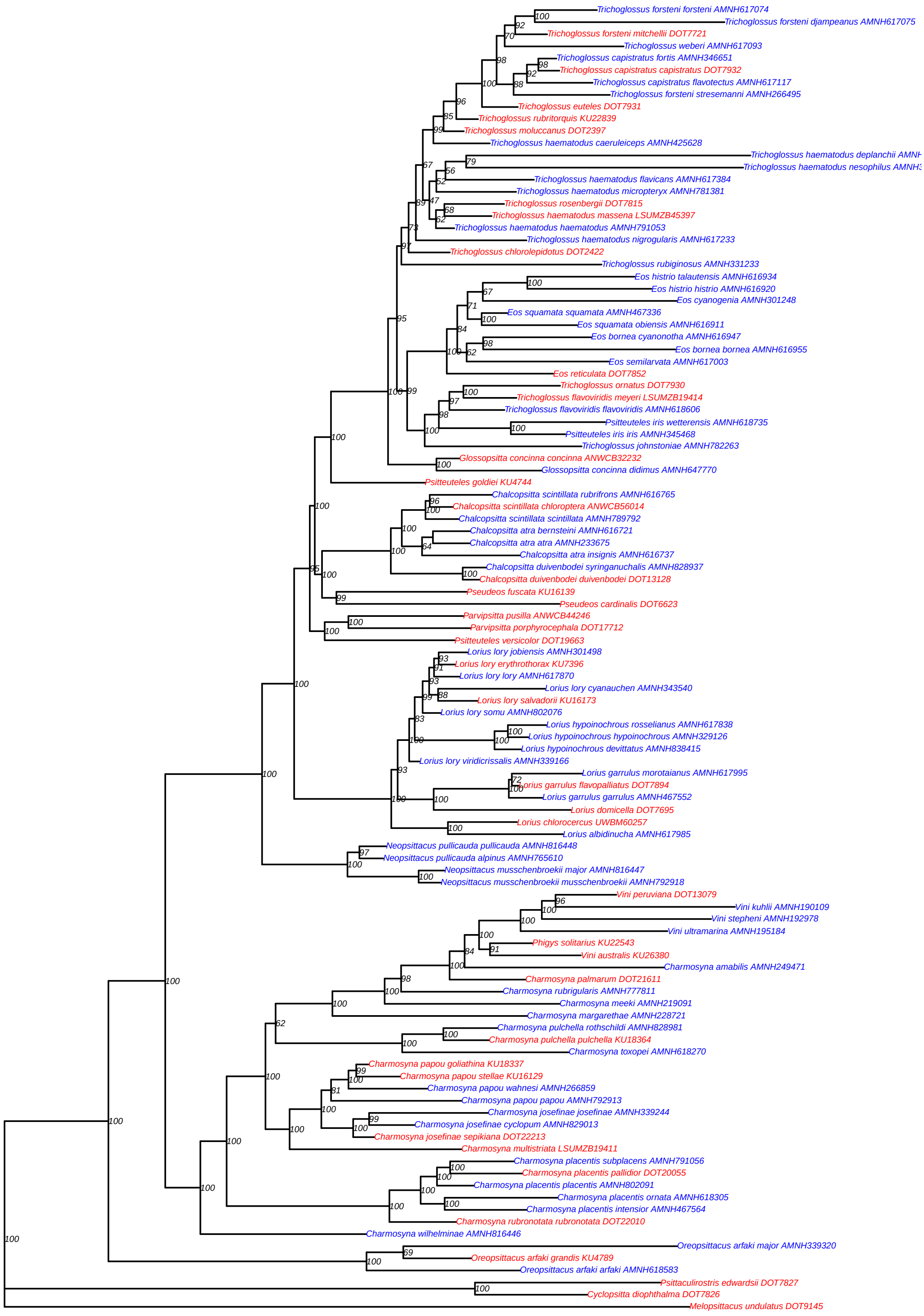

Fig. S11J) Low Coverage: 90% Data Completeness

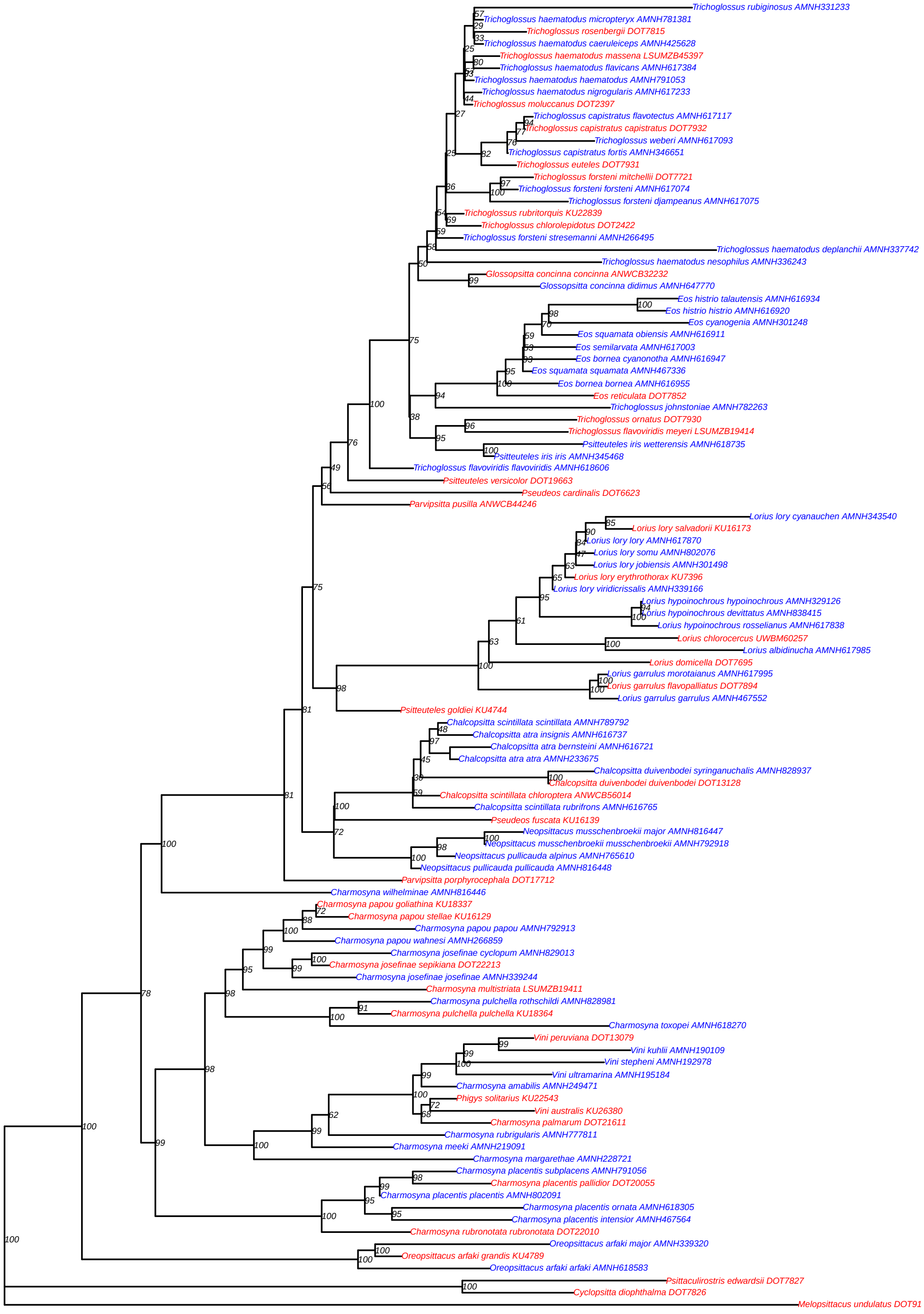

Fig. S11K) Low Coverage: 100% Data Completeness
